# The Connection Between Lipid Metabolism in the Heart and Liver of Wuzhishan Pigs

**DOI:** 10.3390/biom15071024

**Published:** 2025-07-16

**Authors:** Yuwei Ren, Feng Wang, Ruiping Sun, Xinli Zheng, Yanning Lin, Zhe Chao

**Affiliations:** Key Laboratory of Tropical Animal Breeding and Disease Research, Institute of Animal Science and Veterinary Medicine, Hainan Academy of Agricultural Sciences, Haikou 571100, China; renyuwei@hnaas.org.cn (Y.R.);

**Keywords:** pigs, lipid metabolism, balance, correlation, antioxidation

## Abstract

Lipid metabolism is critical for the physiological activities of signal transduction, metabolic regulation, and energy provision, and Wuzhishan (WZS) pigs are a promising animal model for studying human diseases. However, lipid metabolites in the heart and liver of WZS pigs are indistinct. In this study, we detected gene expression, blood biochemical parameters, and metabolic profiles of hearts and livers of WZS and Large White (LW) pigs, and analyzed correlations between metabolites. The results showed that the fatty acid metabolic process was present in both the heart and liver, and was more dominant in the liver. Although the expression of lipid absorption-related genes of *CYP7A1* increased in the liver, *CEBPB* levels increased in both the liver and heart; the fatty acid beta-oxidation genes *RXRA* and *ACSS2* also showed increased expression. The quantity of metabolites related to lipid synthesis decreased in the liver, heart, and blood for WZS pigs compared to that of LW pigs, indicating a balance of lipid synthesis and breakdown for WZS pigs. Moreover, the lipid metabolites in the liver and heart exhibited strong correlations with each other and showed similar correlations to blood biochemical parameters, respectively. This study declared the balance of lipid metabolism in both the heart and liver, and identified their connections for WZS pigs.

## 1. Introduction

Lipid metabolism is a critical process for lots of physiological activities, including signal transduction, metabolic regulation, and energy homeostasis [1]. The heart mainly relies on consuming fatty acids to supply oxidative phosphorylation and energy production [2]. The liver, as the largest metabolic tissue, is responsible for nutrient absorption and energy supply [3]. However, although lipids are a critical source for energy production, an imbalance in lipid metabolism within cardiomyocytes may lead to intracellular lipid accumulation, and excess liver lipid deposition can induce diseases, such as hepatic lipogenesis [4], coronary atherosclerotic cardiopathy [5], obesity, and insulin resistance [6].

Lipids in eukaryotes comprise varying categories, including phospholipids, sphingolipids, sterols, cholesterol, and fatty acids [7], which are essential components of cellular membranes [8]. Lipid metabolism is a crucial biochemical process that involves the breakdown, absorption, biosynthesis, catabolism, and storage of lipids. Its functions range from maintaining biological membrane structure and providing energy to facilitating signal transduction, all of which are essential for the growth and development throughout life [9,10].

The balance between lipid synthesis and lipolysis was necessary for the physiological functions of organs. The increasing expression of hepatic farnesoid x receptors (FXRs) and the decreasing expression of cholesterol 7 alpha hydroxylase (*CYP7A1*) contributed to the expression of cholesterol transporters, which led to the development of cholesterol gallstone disease [11]. The expression trend of the *CYP7A1* gene was opposite to that of fibroblast growth factor receptor 4 (*FGFR4*) [12]. Moreover, the activation of hepatic FXRs reduces lipid absorption and lipogenesis by decreasing bile acids [13], and it promotes lipid metabolism balance by inhibiting the expression of *CYP7A1* and *CYP8B1* [14], suggesting that FXRs primarily mediate lipid metabolism by regulating *CYP7A1* expression. In contrast, *CYP7A1* was a rate-limiting enzyme responsible for the breakdown of cholesterol and the synthesis of bile acid [15]. Bile acids contribute to lipid absorption from the diet, while impaired bile acid conjugation and release can stimulate non-alcoholic fatty liver disease [16].

Blood biomarkers reflect the lipid-related indices of tissues, particularly in the context of liver disease [17]. The elevation of alanine transaminase (ALT) and aspartate aminotransferase (AST) was an indicator of liver damage [18], and the AST/ALT ratio was an effective biomarker for the prediction of liver injury [19]. Direct bilirubin (DBIL) levels exhibited a significant negative correlation with triglyceride (TG) and total cholesterol (TC), indicating that DBIL plays a protective role against high levels of TC and TG [20]. Moreover, ALT, AST, total bilirubin (TBIL), alkaline phosphatase (ALP), γ-glutamyl transferase (γ-GT), TC, and TG levels showed a positive correlation with patients who had cholecystolithiasis and hepatic steatosis [21]. The high-density lipoprotein (HDL) removes lipid particles from the blood vascular wall by transporting cholesterol to the liver [22], while the low-density lipoprotein (LDL) transports cholesterol from the liver to the blood, and is positively correlated with cardiovascular disease [23]. Importantly, the liver lipid metabolites were correlated to blood metals [24].

Animal models have been used for investigating treatments and vaccines for human diseases for a long time, ranging from small rodents to large mammals. Pigs are more suitable due to their similarities to humans in terms of physiologic, immunologic, and genetic characteristics [25]. WZS pigs are a minipig native to Hainan Province, China, and are considered an ideal animal model for research. Although lipid is an important energy source for the liver and the heart, and the liver is the major tissue for performing lipid oxidation and energy provision, the connection between lipid metabolism in the heart and liver for WZS pigs is unclear. In this study, we identified genes and metabolites related to lipid metabolism in the liver and the heart of WZS and LW pigs, and analyzed the correlations among these metabolites. This study aimed to identify genes and metabolites involved in lipid metabolism in the liver and the heart of WZS and LW pigs, and to provide insights into the metabolic crosstalk between these two tissues.

## 2. Materials and Methods

### 2.1. Sample Collection

WZS pigs (n = 6) and LW pigs (n = 6), all healthy in appearance and 4-month-old (3 sows and 3 boars per breed), were purchased from the Nongkenhongmu Agricultural Development Company (Qiongzhong, Haina, China). All the pigs were vaccinated against foot-and-mouth disease, and injected with triple vaccines against three diseases (swine fever, swine erysipelas, swine Pasteurellosis) before purchase. Both WZS and LW pigs were farmed with free feed and water for a week before slaughtering. As all the pigs were piglets and the piglet compound feed contained crude protein (≥16%), fiber (≤6%), crude ash (≤7%), lysine (≥1.1%), calcium (0.6–1.2%), total phosphorus (≥0.4%), sodium chloride (0.3%−0.8%), and water (≤13%). After the adaptive phase, the pigs had their food withdrawn for 24 h and were then transferred to the slaughterhouse at the Yongfa pig experimental facility (Chengmai, Hainan, China). The pigs were stunned via electric shock and exsanguinated, flushed, and split without consciousness. The heart and liver tissues were collected, quickly frozen in liquid nitrogen, and stored at –80 °C before RNA extraction and metabolite extraction.

### 2.2. RNA-Sequencing

Heart and liver tissues from three WZS and three LW pigs (2 sows and 1 boar per breed) were collected for total RNA extraction using an RNA Extraction kit (Qiagen, Cat. No./ID: 74104). RNA libraries were prepared and subjected to RNA-seq on the Illumina Nova 6000 platform by Novogene Corporation (Beijing, China), yielding approximately ~6 Gb of data per sample. Raw data were filtered using fastp to remove adapters and low-quality reads. Clean reads were mapped to the WZS assembly genome (European Nucleotide Archive database, accession number: GCA_965154145.1) using HISAT2 v2.2.1 with default parameters, and FPKM were calculated using StringTie v2.2.1 [26]. Differentially expressed genes (DEGs) were identified using DESeq2 v1.48.1 [27] with thresholds of |log_2_ (foldchange)| ≥ 1, and *p*-value ≤ 0.05. The annotated expressed genes were used for the selection of cardiac genes. The functional enrichment of DEGs was arranged using the R package (version R-4.4.0) clusterProfiler v4.0 [28], and the results were statistically adjusted by *p*-value ≤ 0.05. Protein–protein interaction (PPI) networks were constructed using STRING (https://cn.string-db.org, accessed on 20 May 2025).

### 2.3. Metabolome Analysis

#### 2.3.1. Metabolite Extraction from Tissues

Tissues (100 mg) of hearts and livers from three WZS and LW pigs for each were ground with liquid nitrogen, and the homogenate was resuspended with prechilled 80% methanol by vortex. The homogenate mixed with methanol was incubated on ice for 5 min and then centrifuged at 15,000× *g* at 4 °C for 20 min. The supernatant was diluted to a final concentration with LC-MS grade water and 53% methanol. The samples were subsequently transferred to clean Eppendorf tubes and then centrifuged at 15,000× *g* at 4 °C for 20 min [29], and the liquid supernatant was extracted for LC-MS analysis.

#### 2.3.2. LC-MS Analysis

The LC–MS analysis workflow was displayed in the Supplementary Figure S1. LC–MS was performed using a Vanquish UHPLC system (ThermoFisher, Dreieich, Germany, Chelmsford, MA, USA) coupled with an Orbitrap Q ExactiveTM HF mass Spectrometer (ThermoFisher, Germany, San Jose, CA, USA), which was conducted by Novogene Co., Ltd. (Beijing, China) [30]. Samples prepared in the last step were injected into a Hypersil Goldcolumn (100 × 2.1 mm, 1.9 μm) using a 12-min linear gradient at a flow rate of 0.2 mL/min. The eluents for the positive and negative polarity modes were 0.1% formic acid in water and methanol. The reagent was set as follows: 2% methanol, 1.5 min; 2–85% methanol, 3 min; 85–100% methanol, 10 min; 100–2% methanol, 10.1 min; 2% methanol, 12 min. The quality control (QC) samples used as standard reference were prepared by mixing all the samples in equal volumes. Q ExactiveTM HF mass spectrometer spectrometric signals of the samples were collected from positive and negative ion scanning modes with the conditions as voltage of 3.5 kV, capillary temperature of 320 °C, sheath gas flow rate of 35 psi, and aux gas flow rate of 10 L/min and heater temperature of 350 °C, S-lens RF level of 60 [31].

#### 2.3.3. Data Processing and Metabolite Identification

The raw data files generated by LC-MS were processed using Compound Discoverer 3.3 (version CD3.3, ThermoFisher) to obtain main parameters, including peak area, actual mass tolerance of 5 ppm, signal intensity tolerance of 30%, and minimum intensity. Peak intensities were normalized to the total spectral intensity [32]. The normalized data was used to predict the molecular formula according to additive ions, molecular ion peaks, and fragment ions. The normalized peaks were matched to the databases of mzCloud (https://www.mzcloud.org/, accessed on 15 March 2025), mzVault v2.1, and MassList [33], yielding accurate qualitative and relative quantitative metabolite information. Statistical analyses were performed using R (version R-3.4.3). The sample with a relative standard deviation (RSD), more than 30% of the QC samples was removed, and the remaining samples were finally identified and quantified [34]. The raw data was converted to a format by MSConvert embedded with ProteoWizard [35], and submitted to the National Genomics Data Center (NGDC) (https://ngdc.cncb.ac.cn/).

#### 2.3.4. Metabolites Analysis

These metabolites were annotated using the functional databases of KEGG (https://www.genome.jp/kegg/pathway.html, accessed on 25 March 2025), HMDB (https://hmdb.ca/metabolites, accessed on 25 March 2025), and LIPIDMaps (http://www.lipidmaps.org/, accessed on 25 March 2025). Principal component analysis (PCA) and Partial least squares discriminant analysis (PLS-DA) were performed by the R function prcomp and R (version R-4.4.0) packages mixOmics v6.32.0 [36]. The Student’s *t*-test was used to calculate the statistical significance (*p*-value), and metabolites with VIP ≥ 1, *p*-value ≤ 0.05, and log_2_|fold change| ≥ 1 were considered to be differential metabolites between treatment and control groups.

### 2.4. Blood Biochemical Parameters Test

The blood samples from 12 pigs (6 WZS and LW pigs for each) were centrifuged at 3000 rpm at 4 °C, and the liquid supernatant was extracted. This supernatant was then added to the corresponding kits with 10 μL for each hole, and each sample was repeated three times. The kits included alanine transaminase (ALT) kit (S03030, Leidu, Shenzhen, China), aspartate aminotransferase (AST) kit (S03040, Leidu, Shenzhen, China), alkaline phosphatase (ALP) kit (S03038, Leidu, Shenzhen, China), γ-glutamyl transferase (γ-GT) kit (S03031, Leidu, Shenzhen, China), Direct bilirubin (DBIL) kit (C119, Changchunhuili, Changchun, China), total bilirubin (TBIL) kit (C120, Changchunhuili, Changchun, China), total bile acid (TBA) kit (S03074, Leidu, Shenzhen, China), triglyceride (TG) kit (S03027), cholesterol (CHO) kit (S03042, Leidu, Shenzhen, China), high-density lipoprotein (HDL) kit (S03025, Leidu, Shenzhen, China), low-density lipoprotein cholesterol (LDL) kit (S03029, Leidu, Shenzhen, China). All the biochemicals were detected using an automatic biochemical analyzer (Chemray 800, Shenzhen Leidu Life Technology, Shenzhen, China).

### 2.5. Histological Examination

Tissues of the heart and liver from six WZS and LW pigs for each were selected for slice preparation and staining. The longest muscle tissues from the WZS pigs and the LW pigs collected in step 2.1 were treated with 4% paraformaldehyde, then trimmed, embedded, sectioned, stained (with hematoxylin-eosin (abbreviated as HE), carmine red staining), sealed, and examined. Only qualified paraffin sections were selected for imaging. Hematoxylin is basic, making the chromatin in the cell nucleus and the ribosomes in the cytoplasm appear purple-blue. At the same time, eosin is acidic, causing the cytoplasm and extracellular matrix to appear red. Cell morphology was analyzed using the Pannoramic MIDI II Digital Scanner (3DHISTECH, H-1141 Budapest, Öv u. 3., Hungary).

## 3. Results

### 3.1. Gene Expressions

**Figure 1:**
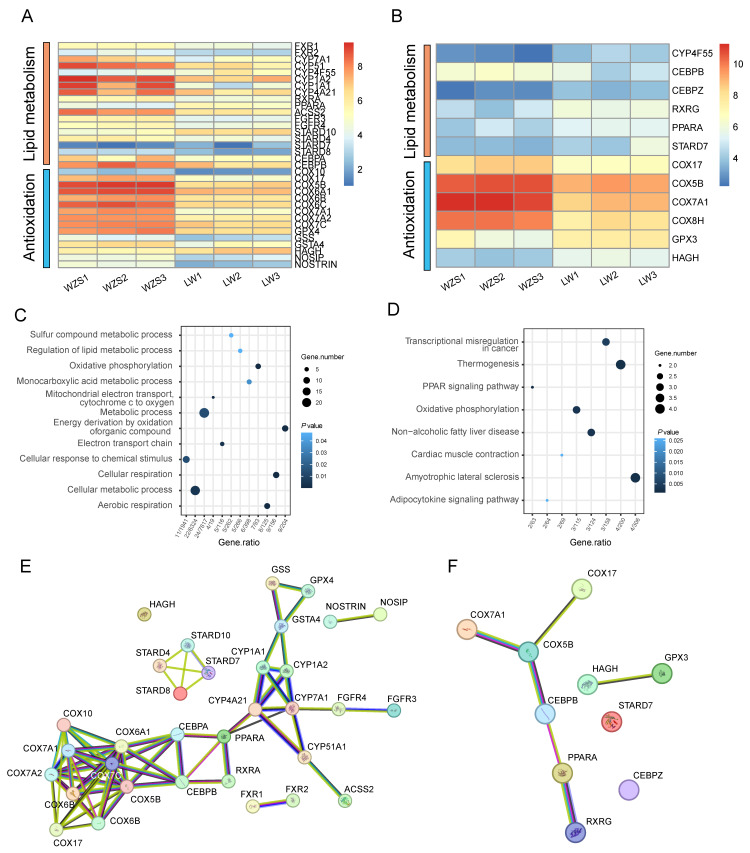
The DEGs related to lipid metabolism of the liver and the heart of pigs. (**A**) The 46 DEGs of liver from WZS and LW pigs. (**B**) The 41 DEGs of the heart from WZS and LW pigs. (**C**,**D**) The GO enrichment of liver and the heart DEGs. (**E**,**F**) The primary PPI functions of the liver and the heart DEGs.

A total of 10,667 and 9907 expressed genes (FPKM > 1) were detected in the liver of WZS and LW pigs, including 3226 DEGs. DEGs were functionally enriched, and 34 DEGs were involved in the top functional terms, which were categorized into two main areas: lipid metabolism and antioxidation (Figure 1A, Supplementary Table S1). Lipid metabolism included bile acid receptors genes (*FXR1*, *FXR2*), retinoid X receptor genes (*RXRA*), peroxisome proliferator-activated receptor alpha (PPARA), acetyl-CoA synthetase 2A (*ACSS2*), CCAAT/enhancer (*CEBPA*, *CEBPB*), fibroblast growth factor receptors (*FGFR3*, *FGFR4*), StAR-related lipid transfer domain containing (*STARD10*, *STARD4*, *STARD8*). In addition, lipid metabolism is a redox reaction that inevitably generates oxidative stress. The electron transport gene was cytochrome c oxidase copper chaperone (COX), and antioxidant genes included nitric oxide synthases (*NOSIP*, *NOSTRIN*), glutathione peroxidase 4 (*GPX4*), glutathione synthetase (*GSS*), glutathione S-transferase A4 (*GSTA4*), and hydroxyacylglutathione hydrolase (*HAGH*). Furthermore, 10,933 and 10,287 expressed genes (FPKM > 1) were detected in WZS and LW pigs’ hearts, including 2663 DEGs, and 12 DEGs were enriched in the cardiac lipid metabolism (Figure 1B, Supplementary Table S2).

In addition, gene expression was different between the heart and liver in WZS and LW pigs. In liver tissue, the lipid-related genes *FXR1*, *FXR2*, *CYP7A1*, *CYP51*, *CEBPA*, *CEBPB*, *RXRA, ACSS2* and FGFRs increased, while *PPARA* and *STARD10* decreased (Figure 1A, Supplementary Table S1). The antioxidation-related genes of nitric oxide synthases (NOSs), COXs, GSS, GPXs, and *GSTA4* increased, while *HAGH* decreased in WZS pigs compared to those in LW pigs. In heart tissue, except for *CEBPB*, the expression of other lipid metabolism genes decreased in WZS pigs compared to those in LW pigs, while all the COXs increased (Figure 1B, Supplementary Table S2).

Furthermore, although the functional enrichment analysis revealed similarities between the heart and liver, the primary functions in the two tissues were different. The lipid metabolic process was presented in both the heart and liver, with a predominant enrichment in the liver (Figure 1C, Supplementary Table S3), while cardiac muscle contraction was specifically enriched in the heart (Figure 1D, Supplementary Table S4). Notably, the network primarily consisted of three parts: lipid metabolism genes (*FXR1*, *FXR2*, *PPARA*, *RXRA*, and *CYP7A1*), cytochrome c oxidase copper chaperone, and glutathione S-transferase. The lipid metabolism genes were located in the hub to connect the antioxidation genes (Figure 1E), and COX5B connected the CEBPB and *PPARA* in the heart (Figure 1F), which suggested the connection between lipid metabolism and antioxidation.

### 3.2. Blood Biochemical Parameters Analysis

**Figure 2:**
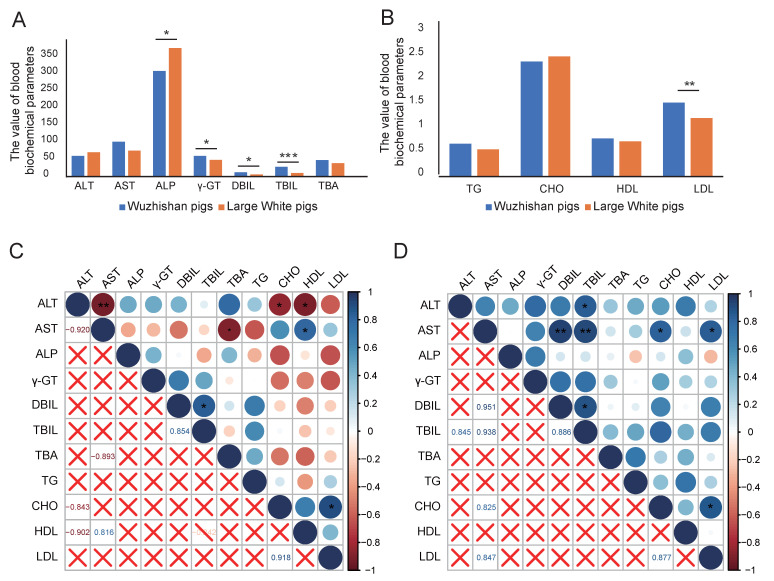
The blood biochemical parameters related to lipid metabolism of pigs. (**A**,**B**) The blood biochemical parameters of WZS and LW pigs. (**C**,**D**) The correlations of blood biochemical parameters in WZS and LW pigs, respectively. The different color blocks in the legend represented the *p*-value ranging from −1 to 1. The symbol “×” indicates that the correlation value was not statistically significant (*p*-value > 0.05). “*” indicates *p*-value ≤ 0.05; “**” indicates *p*-value ≤ 0.01; “***” indicates *p*-value ≤ 0.001.

The blood biochemical parameters were detected to identify their associations with lipid metabolism. The quantity of ALP, γ-GT, DBIL, TBIL, and LDL was significantly different between WZS and LW pigs. The ALP was lower, while other biochemical parameters were higher in WZS pigs than those in LW pigs (Figure 2A,B, Supplementary Table S5). In WZS pigs, AST was negatively correlated with ALT, CHO, and HDL, while AST was positively correlated with HDL (Figure 2C). In LW pigs, AST was positively correlated to CHO, LDL, DBIL, and TBIL. In both WZS and LW pigs, TBIL was positively correlated with DBIL, and CHO was positively correlated with LDL (Figure 2D). Therefore, the blood biochemical parameters of AST, ALT, TBIL, DBIL, LDL, and CHO were correlated with lipid metabolites and reflected the lipid metabolism in the liver and the heart tissue.

### 3.3. Metabolite Analysis of the Liver and the Heart

**Figure 3:**
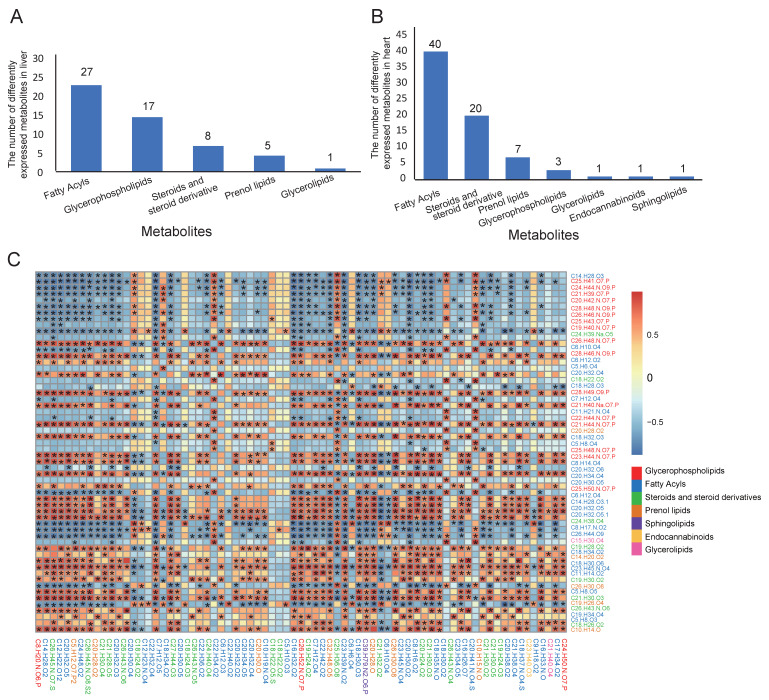
The metabolites related to lipid metabolism show different expressions in the liver and the heart of WZS and LW pigs. (**A**) The categories and quantity of 58 metabolites in the liver. (**B**) The categories and quantity of 73 metabolites in the heart. (**C**) The correlations between metabolites in the liver and the heart are shown in the figure. The different color blocks above in the legend represent correlation values ranging from −1 to 1, while the color blocks below represent different categories of metabolites. “*” indicates *p* value ≤ 0.05 for the correlations.

The metabolites were different in the liver and the heart between WZS and LW pigs. A total number of 58 formulas related to lipid metabolism showed different expressions in the liver between WZS and LW pigs (Supplementary Table S6), which primarily included fatty acyl and glycerophospholipids, a few prenol lipids, and steroids and steroid derivatives (Figure 3A). Importantly, most of the fatty acyls decreased, and most of the glycerophospholipids increased, while prenol lipids decreased in WZS pigs compared to those of LW pigs. The metabolites in the liver suggested that the level of lipid synthesis was lower in the liver of WZS pigs than that of LW pigs. Moreover, a total of 73 formulas related to lipid metabolism showed different expressions in the heart between WZS and LW pigs (Supplementary Table S7). Unlike the liver, the major metabolites in the heart were fatty acyls, steroids, and steroid derivatives, along with a few glycerophospholipids (Figure 3B). Like most of the fatty acids, steroids, and steroid derivatives decreased in the heart of WZS pigs than those in the LW pigs, the level of lipid synthesis was also lower in the heart of WZS pigs compared to LW pigs. Furthermore, the metabolites in the liver and the heart were correlated with each other (Supplementary Tables S8 and S9). Most of the liver glycerophospholipids were negatively correlated to heart fatty acids, steroids, and steroid derivatives. In contrast, most of the liver fatty acids, steroids, and steroid derivatives were positively correlated with heart metabolites (Figure 3C).

### 3.4. The Correlations Between Blood Biochemical Parameters and Tissue Metabolites

**Figure 4:**
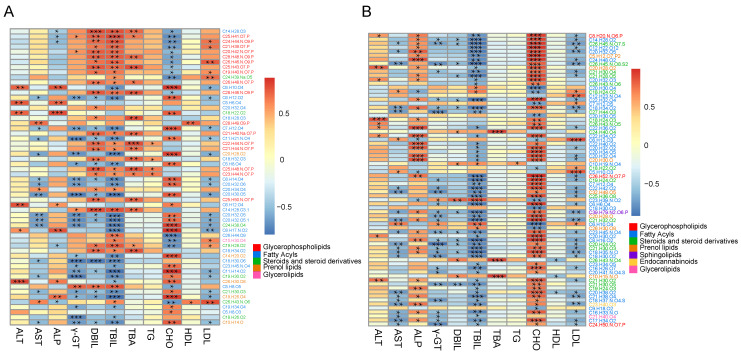
The correlations between blood biochemical parameters and metabolites of the liver and the heart. (**A**) The correlations between blood biochemical parameters and 58 liver metabolites. (**B**) The correlations between blood biochemical parameters and 73 metabolites of the heart. The different color blocks in the legend above represent correlation values ranging from −1 to 1, “*” indicates *p*-value ≤ 0.05; “**” indicates *p*-value ≤ 0.01; “***” indicates *p*-value ≤ 0.001, while the different color blocks below represent different categories of metabolites.

The correlations between blood biochemical parameters and liver metabolites were similar to those between blood biochemical parameters and the heart. In the liver, the ALP and CHO were negatively correlated with glycerophospholipids, and positively correlated with most of the fatty acyl and prenol lipids. In contrast, AST, γ-GT, DBIL, TBIL and LDL were positively correlated to most of the glycerophospholipids, and negatively correlated to most of the fatty acyls and prenol lipids; CHO was positively correlated to most of the fatty acyls, and negatively correlated with most glycerophospholipids; ALT was positively correlated to fatty acyl in liver (Figure 4A, Supplementary Tables S10 and S11). Furthermore, the blood biochemical parameters were also associated with heart metabolites (Figure 4B, Supplementary Tables S12 and S13). The AST, γ-GT, DBIL, TBIL, and LDL were negatively correlated, while ATL, ALP, TBA, and CHO were positively correlated with most of the fatty acyls, steroids, and steroid derivatives in the heart.

### 3.5. Histological Examination Analysis

**Figure 5:**
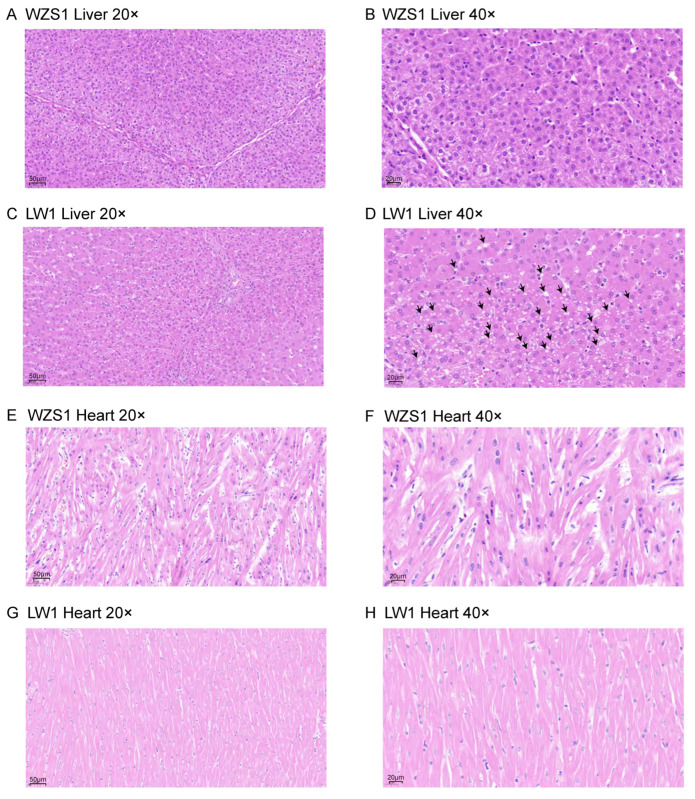
The HE-stained slice was prepared from a pig’s liver. (**A**,**B**) The HE staining slice of liver with 20× and 40× magnification from one WZS pig. (**C**,**D**) The HE staining slice of liver with 20× and 40× magnification from one LW pig. The black arrows in (**D**) pointed to vacuoles, which presented as fatty liver. (**E**,**F**) The HE staining slice of the heart with 20× and 40× magnification from one WZS pig. (**G**,**H**) The HE staining slice of the heart with 20× and 40× magnification from one LW pig. The plotting scales of 20× and 40× magnification were 50 μm and 20 μm, respectively.

Notably, two of the six LW pigs exhibited small vacuoles in the liver upon HE staining, a finding not observed in any of the WZS pigs (Figure 5A–D, Supplementary Figures S2 and S3), and these vacuoles were diagnosed as indicative of fatty liver. However, the heart tissues did not show obvious symptoms related to excess lipid storage (Figure 5E–H, Supplementary Figures S4 and S5). In summary, the comprehensive results, including decreased metabolites of fatty acyls, steroids and steroid derivatives in liver and the heart for WZS pigs than that in LW pigs, and their positive correlations with CHO, as well as the mild fatty liver in LW pigs but not in WZS pigs, demonstrated the levels of lipid synthetase and lipid storage were lower in liver and the heart of WZS pigs than that in LW pigs.

## 4. Discussion

The different gene expressions and metabolites uncovered the connections between lipid metabolism in the heart and liver for WZS pigs. The lipid metabolic process was active in both the heart and liver, and was predominantly enriched in the liver, while cardiac muscle contraction was specifically enriched in the heart. Although the expression of *CYP7A1* and *CEBPA* is primarily related to lipid absorption and storage, increased in the liver, and *CEBPB* increased in both the liver and the heart, the fatty acid beta-oxidation gene *ACSS2* also increased in the liver, indicating that both lipid synthesis and oxidation levels increased in the liver of WZS pigs. Furthermore, most of the metabolites associated with lipid synthesis were decreased in the liver and the heart of WZS pigs compared to those in LW pigs, which indicated a balance between lipid synthesis and breakdown.

The functional enrichment revealed two major parts, including lipid metabolism and antioxidation, in both the liver and the heart. The key genes involved in lipid metabolism were FXRs, *CYP7A1*, *CEBPA*, *CEBPB*, *RXRA*, *PPARA*, and *ACSS2.* The FXRs could inhibit lipid absorption and storage by reducing bile acid production, while *CYP7A1* is an important gene involved in the regulation of bile acid biosynthesis. The FXR activation was accompanied by a decrease in the expression of *CYP7A1* [11], and an increase in the expression of *FGFR4* and *CYP27A1* [12,14]. However, in this study, the expressions of *FXR1*, *FXR2*, *CYP7A1*, and *FGFR4* increased in the liver of WZS pigs compared to LW pigs, and the probable explanation was that the increasing rate of FXRs was too weak to inhibit *CYP7A1* gene expression in WZS pigs. Moreover, lipid droplet accumulation led to the dysfunction of macrophage-specific autophagy by inducing macrophage foam cell formation, which was responsible for the pathogenesis of atherosclerosis. Meanwhile, *CEBPB* contributed to lipid accumulation in the macrophage [37]. In this study, *CEBPB* showed higher expression in both the heart and the liver for WZS compared to LW pigs, suggesting that the CEBPs enhanced lipid absorption in both the heart and the liver for WZS pigs. In addition, *PPARA* is a regulator responsible for lipid catabolism and inhibiting lipid storage. The inhibition of the *PPARA* gene could improve lipid profiles by enhancing fatty acid oxidation [38], and its dysfunction may cause obesity and insulin resistance [39]. *RXRA* binds with PPARs to regulate lipid storage, adiponectin production, fat deposition, and inflammation. *RXRA* promotes cholesterol efflux from macrophages [40], which is beneficial for preventing atherosclerosis [41]. *ACSS2* was able to promote fatty acid oxidation by activating *CPT1A* [42]. The loss of hepatic *ACSS2* exacerbates steatosis and reduces the expression of fatty acid oxidation-related genes [43]. In this study, the expression of *PPARA* decreased in both the liver and the heart of WZS pigs. Meanwhile, *RXRA* and *ACSS2* increased in the liver, particularly with the high expression level of *ACSS2* in WZS pigs, suggesting that *ACSS2* was probably the primary gene responsible for regulating lipid oxidation. As a result, although the gene expressions of *CYP7A1* and *CEBPB* mediating lipid absorption increased, the genes *RXRA* and *ACSS2* regulating fatty acid beta-oxidation also increased in the liver for WZS pigs than that in LW pigs; therefore, the synchronous processes of lipid absorption and fatty acid oxidation maintained lipid homeostasis in the liver for WZS pigs.

Furthermore, the antioxidant ability was high in the heart and the liver of WZS pigs. The expressions of NOSs, COXs, and GSTA4 increased in the liver, and COXs increased in both the liver and the heart in WZS pigs compared to those in LW pigs. GPXs have long been utilized as antioxidant enzymes to resist oxidative stress and maintain redox balance [44], while GSTs play crucial roles in cellular detoxification, safeguarding tissues from oxidative damage [45]. Importantly, *GPX4*, GSS, and *GSTA4* showed higher expressions in the liver, and *GPX3* increased in the heart of WZS pigs than in that of LW pigs, indicating glutathione peroxidase-mediated antioxidation was stronger in WZS pigs than in LW pigs. Lipid droplet accumulation in the endothelium suppresses endothelial nitric oxide synthase (eNOS), resulting in the suppression of NO production, elevated blood pressure, endothelial inflammation, and accelerated atherosclerosis [46]. Acute liver failure was related to decreased endothelial NOS isoform and increased reactive oxygen species (ROS) [47]. COXs are the terminal oxidase of the mitochondrial respiratory chain, which is essential for the assembly of the mitochondrial respiratory chain complex [48]. Thus, the increasing expression of COX genes contributed to the energy provision in the heart and the liver for WZS pigs, and the NOSs were protective to the heart and the liver, and the COXs and NOS interacted with each other. Moreover, the fibroblast is a crucial component for cytothesis and cell repair, but it also leads to fibrosis in heart disease. In this study, FGFRs were only expressed in the liver, suggesting that lipid storage and fibrocytes were more abundant in the liver than in the heart. As a result, the NOS, COXs, GPXs, and GSTs genes protected the heart and the liver of WZS pigs by reducing the ROS generated from lipid metabolism, thereby maintaining the balance of lipid-related energy provision.

The lipid-related metabolites in the liver and the heart were also crucial for various functions. The inhibition of liver lipid accumulation in diabetic rats could rescue metabolic disorders by increasing glycerophospholipids and reducing inflammation [49]. The reduction of fatty acyls was accompanied by attenuated liver fibrosis and upregulated *GPX4* expression [50]. Thus, the increasing expressions of FXRs and *GPX4* genes, and glycerophospholipids in the liver, and the decreasing expressions of fatty acyls, steroids, and steroid derivatives in the heart and the liver for WZS pigs compared to those in LW pigs, indicating that most of the lipid storage was used for oxidation and energy provision in WZS pigs. Notably, most of the glycerophospholipids were detected in the liver, indicating that the liver is the primary tissue for lipid oxidation. Moreover, the vacuoles were only observed in the liver, suggesting that LW pigs were more susceptible to mild fatty liver than WZS pigs.

The metabolites in the heart and the liver showed strong correlations with each other and with blood biochemical parameters. Although the primary metabolites differed in two tissues, the correlation tendencies were similar between blood biochemical parameters and lipid metabolism in the liver and the heart. In detail, the ATL and CHO were negatively correlated with glycerophospholipids and positively correlated with most of the fatty acids in the liver. At the same time, AST, γ-GT, DBIL, TBIL, and LDL showed the opposite correlations. As HDL removed lipid particles from the blood vascular wall by transferring lipid from blood to the liver, LDL performed the opposite function [22,23], which was consistent with the positive correlation between blood LDL and CHO, and the negative correlation between blood LDL and liver lipid synthesis in this study. Although the main metabolite were fatty acyl, steroids, and steroid derivatives in the heart, their correlations to blood biochemical parameters were similar to that between liver fatty acyl and blood biomarkers, which indicated that the blood biochemical parameters, glycerophospholipids, fatty acyl, steroids, and steroid derivatives could connect the lipid metabolism between heart and liver. Notably, the strong interlinkage between the two tissues was indispensable for biological functions, as the liver performed lipid oxidation for energy provision, and lipid is the predominant energy source for the heart. However, the detailed patterns in the balance model for the correlations in lipid metabolism between the liver and the heart were complicated, which requires an in-depth study.

## 5. Conclusions

In this study, gene expression and metabolite analysis identified connections between lipid metabolism in the liver and heart of WZS and LW pigs. When comparing the two pig breeds, the lipid metabolic process existed in both the heart and the liver, and was predominantly enriched in the liver. In summary, although the lipid absorption-related genes *CYP7A1* and *CEBPB* increased, the fatty acid beta-oxidation genes *RXRA* and *ACSS2* also increased in the liver of WZS pigs compared to those in LW pigs. Moreover, the decreasing expressions of fatty acyls, steroids, and steroid derivatives in the liver and the heart for WZS pigs than those of LW pigs, as well as the mild fatty liver in LW pigs but not in WZS pigs, identified the greater ability to maintain balance of lipid synthesis and breakdown for WZS pigs compared to LW pigs. This study investigated the genes and metabolites of lipid metabolism involved in the liver and the heart, and demonstrated the homeostasis in the connections between lipid metabolism in these two tissues.

## Data Availability

The RNA-seq data in this study were submitted to the NCBI database (PRJNA1271259), and metabolome sequencing data were submitted to the National Genomics Data Center (PRJCA040474).

## Supplementary Materials

The following supporting information can be downloaded at: https://www.mdpi.com/article/10.3390/biom15071024/s1, Figure S1: The workflow of the LC-MS protocol; Figure S2: The HE staining figures of the livers of WZS pigs; Figure S3: The HE staining figures of the livers of LW pigs; Figure S4: The HE staining figures of the hearts of WZS pigs; Figure S5: The HE staining figures of the hearts of LW pigs; Table S1: The gene expressions in the liver of WZS and LW pigs; Table S2: The gene expressions in the heart of WZS and LW pigs; Table S3: The GO enrichment of liver DEGs related to lipid metabolism; Table S4: The GO enrichment of heart DEGs related to lipid metabolism; Table S5:The blood biochemical parameters related to lipid metabolism; Table S6: The lipid metabolites in liver of WZS and LW pigs; Table S7: The lipid metabolites in heart of WZS and LW pigs; Table S8: The correlations of metabolites related to lipid in liver and the heart; Table S9: The significance of metabolites related to lipid in liver and the heart; Table S10: The correlations between blood biochemical parameters and liver metabolites; Table S11: The significance of correlations between blood biochemical parameters and liver metabolites; Table S12: The correlation between blood biochemical parameters and heart metabolites; Table S13: The significance of correlation between blood biochemical parameters and heart metabolites.

## Abbreviations

WZS pigs: Wuzhishan pigs; LW pigs: Large White pigs; DEG: differentially expressed genes; FXRs: farnesoid x receptors; CYP7A1: cholesterol 7 alpha hydroxylase; FGFR4: fibroblast growth factor receptor 4; CYP8B1: cytochrome P450 8B1; RXRA: retinoid X receptor genes; PPARA: peroxisome proliferator activated receptor alpha; ACSS2: acetyl-CoA synthetase 2A; CEBP: CCAAT/enhancer; GPX4: glutathione peroxidase 4; GSS: glutathione synthetase; GSTA4: glutathione S-transferase; HAGH: hydroxyacylglutathione hydrolase; STARD: StAR related lipid transfer domain containing; COX: cytochrome c oxidase copper chaperone; NOS: nitric oxide synthase; eNOS: endothelial nitric oxide synthase; ALT: alanine transaminase; AST: aspartate aminotransferase; DBIL: direct bilirubin; TG: triglyceride; TC: total cholesterol; TBIL: total bilirubin; ALP: alkaline phosphatase; γ-GT: γ-Glutamyl transferase; HDL: high-density lipoprotein; LDL: low-density lipoprotein; TBA: total bile acid.
