# Supplementary material for: The Connection Between Lipid Metabolism in the Heart and Liver of Wuzhishan Pigs
Source: Biomolecules. 2025 Jul 16;15(7):1024. doi: 10.3390/biom15071024 (PMC12292529; doi:10.3390/biom15071024)
Supplement: Supplementary file 1 [file biomolecules-15-01024-s001.zip › biomolecules-3711312-Supplementary Figures and Table.pdf]

Supplementary Materials

Supplementary Figures

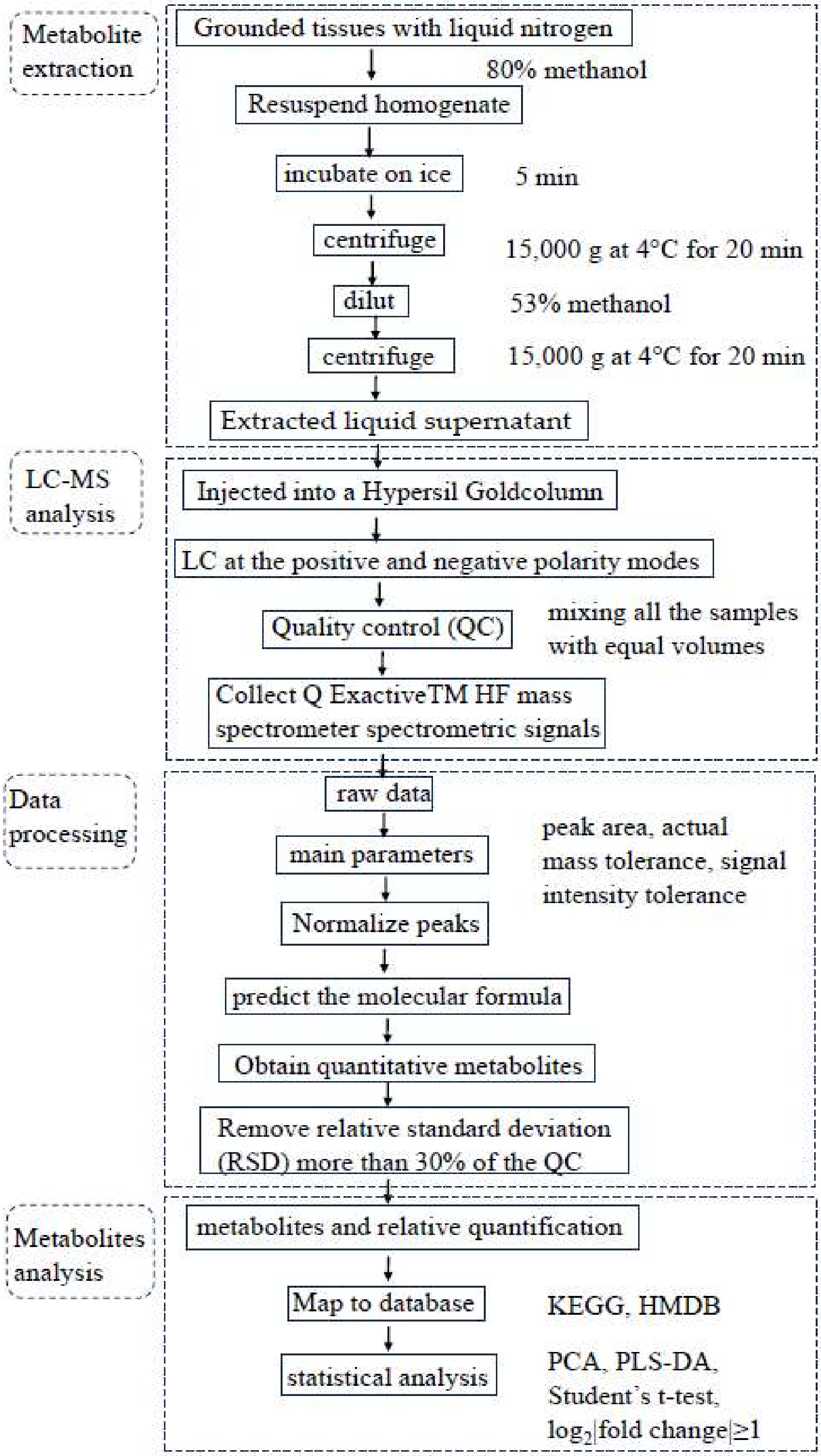

Figure S1 The workflow of the LC-MS protocol.

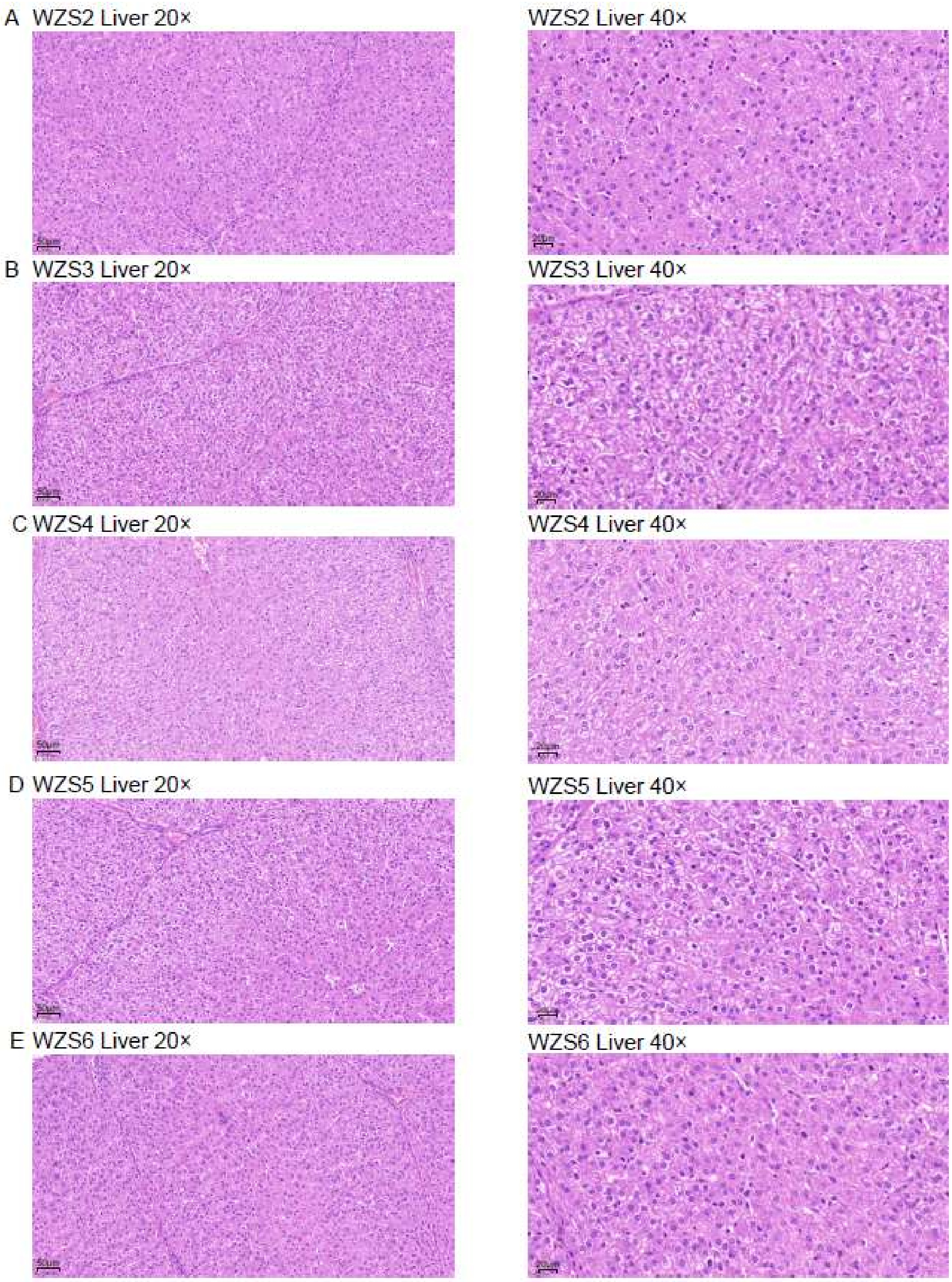

Figure S2 The HE staining figures of the livers of WZS pigs. (A)-(E) The HE staining slice of liver with 20× and 40× magnification from five WZS pigs. The plotting scales of 20× and 40× magnification were 50μm and 20μm, respectively.

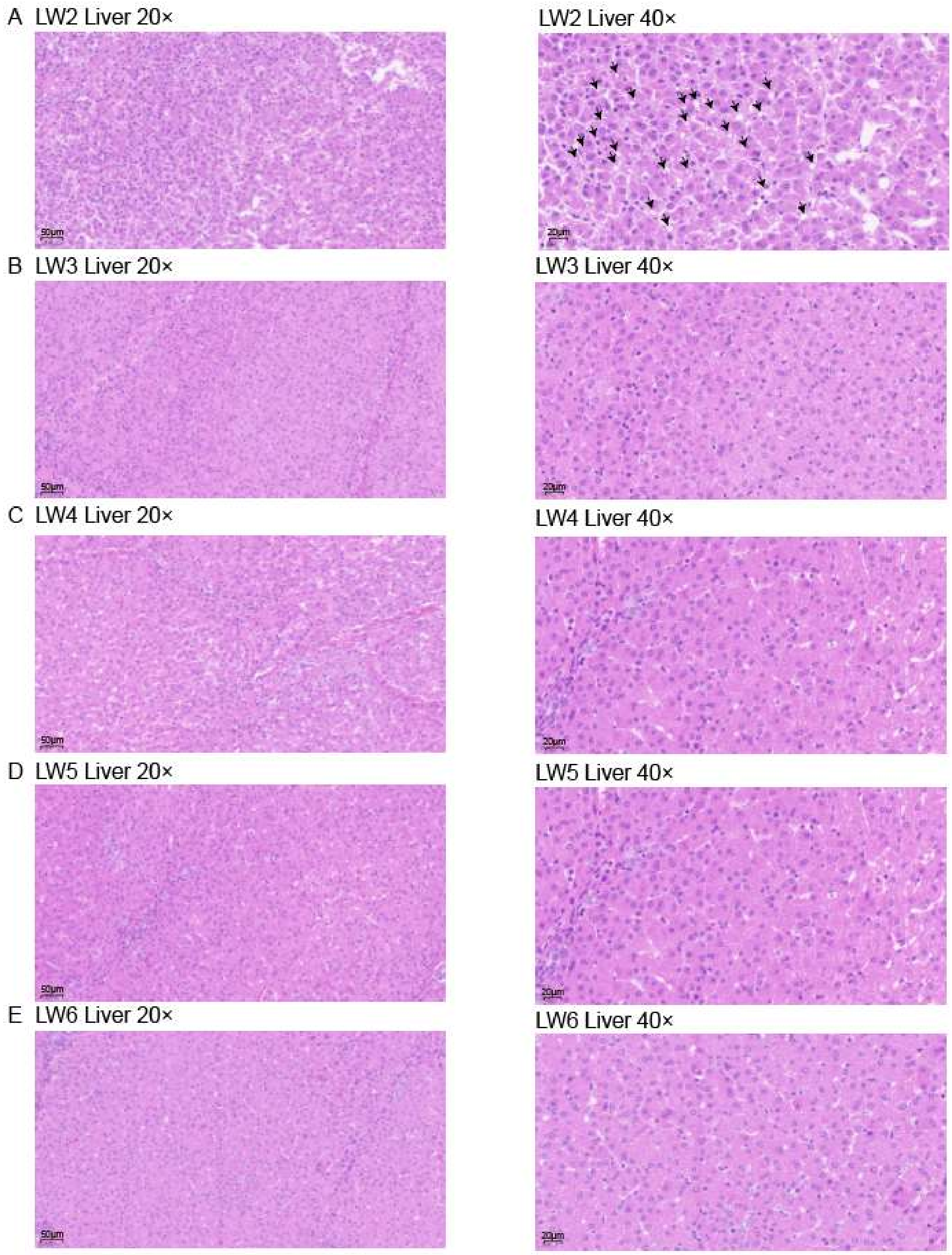

Figure 3 The HE staining figures of the livers of LW pigs. (A)-(E) The HE staining slice of liver with 20× and 40× magnification from five LW pigs. The black arrows on the 40× magnification of **(A)** pointed to vacuoles, which presented as fatty liver. The plotting scales of 20× and 40× magnification were 50μm and 20μm, respectively.

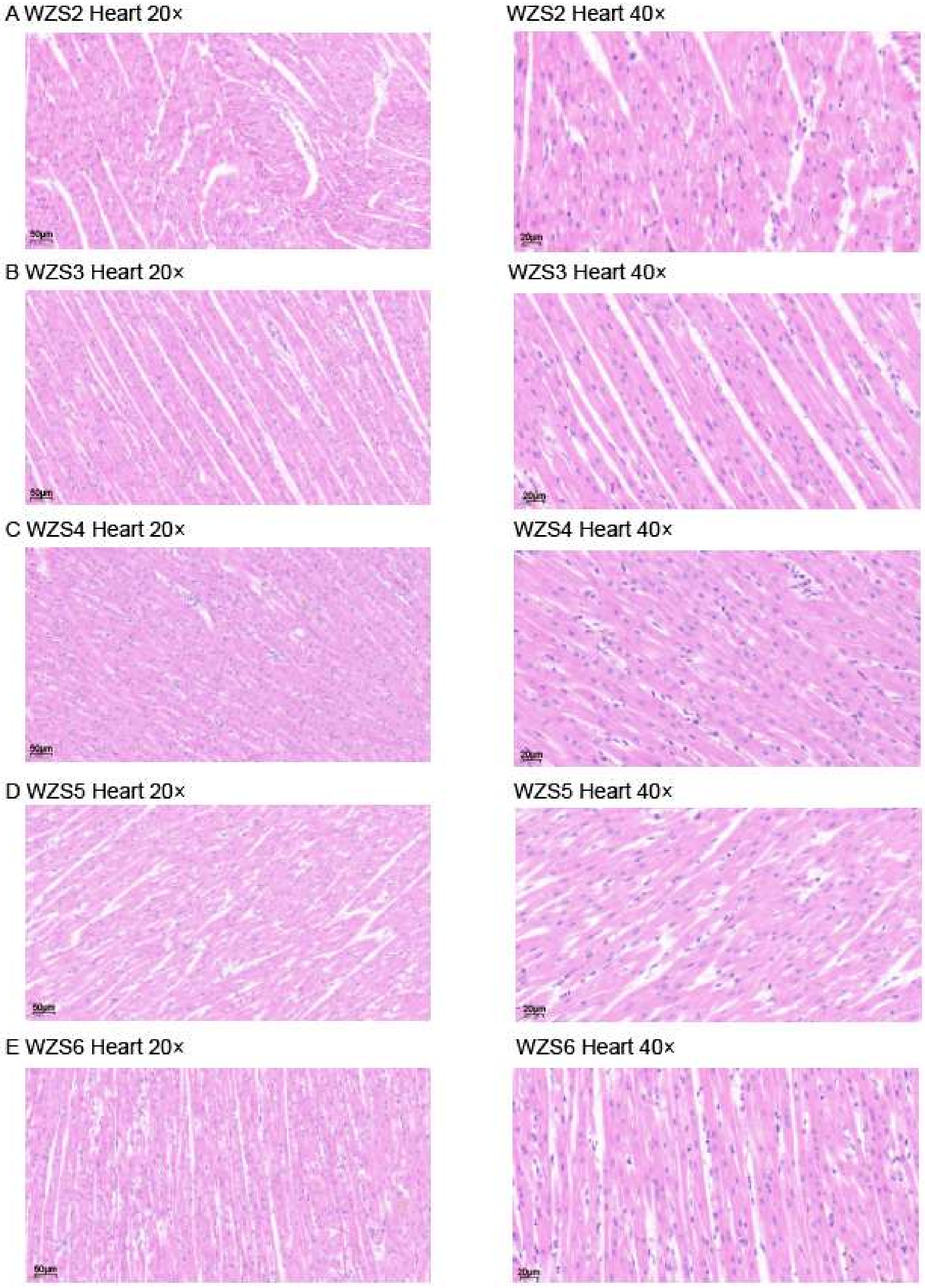

Figure S4 The HE staining figures of the hearts of WZS pigs. (A)-(E) The HE staining slice of heart with 20× and 40× magnification from five WZS pigs. The plotting scales of 20× and 40× magnification were 50μm and 20μm, respectively.

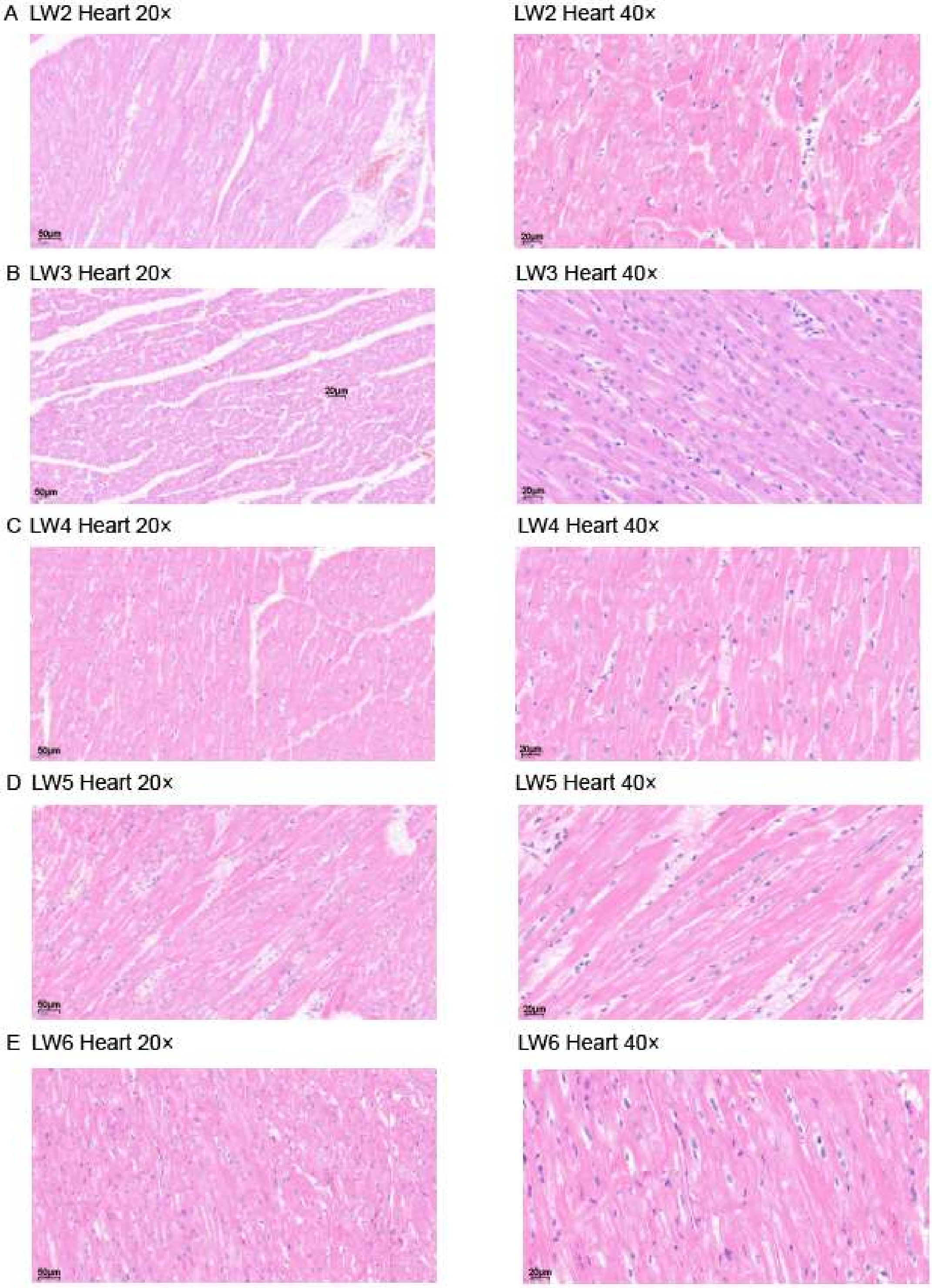

Figure S5 The HE staining figures of the hearts of LW pigs. (A)-(E) The HE staining slice of heart with 20× and 40× magnification from five LW pigs. The plotting scales of 20× and 40× magnification were 50μm and 20μm, respectively.

Supplementary Tables

Table S1 The gene expressions in the liver of WZS and LW pigs

	Groups	Genes	WZS1	WZS2	WZS3	LW1	LW2	LW3	Log_2 FC	*P*-value
1	Farnesoid X Receptor	*FXR1*	42.2	42.91	42.71	21.93	22.95	17.33	1.04	0.018
2	Farnesoid X Receptor	*FXR2*	12.31	18.92	13.46	7.83	6.83	6.98	1.05	0.044
3	Cytochrome P450 proteins	*CYP7A1*	196.42	116.5	74.68	12.5	34.99	40.18	2.14	0.013
4	Cytochrome P450 proteins	*CYP51*	477.93	341.42	286.11	91.98	103.93	51.12	2.16	6.2E-44
5	Cytochrome P450 proteins	*CYP4F55*	12.73	15.54	20.79	24.42	74.11	37.31	-1.47	4.6E-04
6	Cytochrome P450 proteins	*CYP1A2*	667.98	402.11	487.75	134.66	100.45	173.26	1.93	1.9E-04
7	Cytochrome P450 proteins	*CYP1A1*	542.38	162.05	473.08	24.8	8.1	24.79	4.35	4.9E-12
8	Cytochrome P450 proteins	*CYP4A21*	409.86	140.57	339.84	136.73	59.78	99.05	1.59	0.039
9	CCAAT/ enhancer	*CEBPA*	116.85	39.89	156.49	29.58	36.15	35.91	1.62	0.036
10	CCAAT/ enhancer	*CEBPB*	227.07	393.19	271.95	149.76	72.73	88.77	1.52	0.042
11	fibroblast growth factor receptor	*FGFR3*	46.86	52.52	58.49	15.77	13.67	16.81	1.77	0.001
12	fibroblast growth factor receptor	*FGFR4*	36.64	44.97	48.1	17.89	20.99	25.34	1.01	0.038
13	retinoid X receptor	*RXRA*	39.7	45.19	55.77	19.18	22.56	25.86	1.06	0.001
14	StAR related lipid transfer domain	*STARD10*	24.69	28.31	38.26	101.01	88.52	107.63	-1.70	0.027

15	StAR related lipid transfer domain	*STARD4*	59.52	62.62	42.94	20.62	25.28	16.9	1.39	0.043
16	StAR related lipid transfer domain	*STARD7*	1.6	1.14	1.77	3.66	1.05	5.15	-1.13	0.001
17	StAR related lipid transfer domain	*STARD8*	8.41	7.77	8.94	4.77	2.62	3.06	1.27	0.049
18	nitric oxide synthase	*NOSIP*	21.52	20.54	26.78	6.92	9.34	13.05	1.23	0.016
19	nitric oxide synthase	*NOSTRIN*	24.42	18.74	19.88	3.54	4.35	5.71	2.21	0.001
20	cytochrome c oxidase copper chaperone	*COX10*	6.2	4.48	6.48	1.87	2.15	2.56	1.38	0.049
21	cytochrome c oxidase copper chaperone	*COX17*	145.22	193.64	190.28	31.99	24.28	28.48	2.64	0.001
22	cytochrome c oxidase copper chaperone	*COX5B*	479.56	590.09	576.55	92.06	96.24	122.21	2.41	0.001
23	cytochrome c oxidase copper chaperone	*COX6A1*	440.22	490.71	475.47	162.91	135.91	146.33	1.66	0.001
24	cytochrome c oxidase copper chaperone	*COX6B*	161.56	216.33	239.81	75.02	68.18	69.46	1.54	0.001
25	cytochrome c oxidase copper chaperone	*COX6C*	374.32	465.48	373.71	103.04	101.26	98.06	2.00	0.001
26	cytochrome c oxidase copper chaperone	*COX7A1*	190.62	251.85	236.59	27.56	28.09	30.17	2.98	0.001
27	cytochrome c oxidase copper	*COX7A2*	239.64	230.07	225.4	54.02	60.47	67.48	1.93	0.001

28	chaperone cytochrome c oxidase copper chaperone	*COX7C*	240.87	269.87	275.27	129.46	92.21	96.66	1.30	0.001
29	glutathione peroxidase	*GPX4*	221.27	257.21	302.57	103.86	85.21	93.16	1.47	0.007
30	glutathione synthetase	*GSS*	21.74	13.33	16.83	5.08	7.05	9.29	1.28	0.035
31	glutathione S-transferase	*GSTA4*	74.28	103.74	98.39	19.68	44.74	34.73	1.48	0.048
32	hydroxyacyl glutathione hydrolase	*HAGH*	57.4	44.18	47.23	79.54	127.44	133.05	-1.19	0.048
33	peroxisome proliferator activated receptor alpha	*PPARA*	21.54	14.59	13.72	45.75	40.78	30.78	-1.23	4.43e-13
34	Acyl-CoA Synthetase Short Chain Family Member 2	*ACSS2*	343.03	215.86	224.52	77.32	79.91	59.86	1.85	5.95e-6

Table S2 The gene expressions in the heart of WZS and LW pigs

	Groups	Genes	WZS1	WZS2	WZS3	LW1	LW2	LW3	Log_2 FC	*padj*
1	cytochrome c oxidase copper chaperone	*CYP4F55*	6.67	5.48	3.7	11.85	20.87	15.75	-1.61	0.001
2	CCAAT/ enhancer	*CEBPB*	74.4	91.64	72.83	54.95	17.09	29.28	1.24	0.047
3	CCAAT/ enhancer	*CEBPZ*	4.18	5.88	5.67	13.64	10.52	12.98	-1.24	0.001
4	retinoid X receptor	*RXRG*	23.09	13.51	30.05	66.73	52.08	62.7	-1.45	0.001
5	StAR related lipid transfer domain	*STARD7*	12.16	12.53	10.55	17.85	14.76	57.9	-1.36	0.001

6	cytochrome c oxidase copper chaperone	*COX17*	289.02	393.47	377.86	96.72	101.62	115.99	1.75	0.001
7	cytochrome c oxidase copper chaperone	*COX5B*	1422.41	1668.63	1616.77	542.91	711.75	620.39	1.33	0.027
8	cytochrome c oxidase copper chaperone	*COX7A1*	2440.56	2472.57	1845.16	350.34	488.67	493.72	2.34	0.001
9	cytochrome c oxidase copper chaperone	*COX8H*	1132.52	1157.31	912.76	170.26	322.81	253	2.1	0.001
10	glutathione peroxidase	*GPX3*	141.06	63.73	52.35	168.18	197.51	209.42	-1.16	0.001
11	hydroxyacylglutathione hydrolase	*HAGH*	16.69	14.3	16.31	48.14	56.58	45.9	-1.67	0.001
12	peroxisome proliferator activated receptor alpha	*PPARA*	14.57	30.49	18.05	40.75	45.78	41.22	-1.02	1.41e-13

Table S3 The GO enrichment of liver DEGs related to lipid metabolism

	GO	Description	Gene ratio	Gene number	**Genes**
1	GO:0008152	Metabolic process	24/7817	0.013	HAGH,GPX4,GSS,GSTA4,CYP1A1,CYP1A2,CYP4A21,CYP51A1,ACSS2,CYP7A1,FGFR4,FGFR3,PPARA,RXRA,CEBPB,COX6A1,COX5B,COX7C,COX10,COX7A1,COX7A2,COX6C,COX6B,NOSIP
2	GO:004	Cellular metabolic process	22/6324	0.004	HAGH,GPX4,GSS,GSTA4,CYP1A1,CYP1A2,CYP4A21,ACSS2,CYP7A1,FGFR4,FG

	4237				FR3,PPARA,RXRA,CEBPB,COX6A1,COX5B,COX7C,COX10,COX7A1,COX7A2,COX6C, COX6B
3	GO:0070887	Cellular response to chemical stimulus	11/1941	0.013	FGFR4,FGFR3,CYP7A1,CYP1A1,CYP1A2,GPX4,GSTA4,PPARA,RXRA,CEBPB,CEBPA
4	GO:0015980	Energy derivation by oxidation oforganic compounds	9/204	1.20E-06	COX6A1,COX5B,COX7C,COX10,COX7A1,COX7A2,COX6C,COX6B,CYP1A2
5	GO:0045333	Cellular respiration	9/156	2.34E-06	COX6A1,COX5B,COX7C,COX10,COX7A1,COX7A2,COX6C,COX6B,CYP1A2
6	GO:0009060	Aerobic respiration	8/125	8.99E-06	COX6A1,COX5B,COX7C,COX10,COX7A1,COX7A2,COX6C,COX6B
7	GO:0006119	Oxidative phosphorylation	7/93	4.74E-05	COX6A1,COX5B,COX7C,COX7A1,COX7A2,COX6C,COX6B
8	GO:0032787	Monocarboxylic acid metabolic process	6/398	3.60E-02	CYP1A1,CYP1A2,CYP7A1,PPARA,HAGH,GPX4
9	GO:0019216	Regulation of lipid metabolic process	5/266	0.047	CYP7A1,FGFR4,FGFR3,PPARA,STARD4
10	GO:0022900	Electron transport chain	5/116	3.00E-03	COX6A1,COX5B,COX7C,COX7A1,COX7A2
11	GO:0006790	Sulfur compound metabolic process	5/262	0.047	GSS,GSTA4,GSTO1,ACSS2,HAGH
12	GO:0006123	Mitochondrial electron transport,cytochrome c to oxygen	4/19	1.00E-04	COX5B,COX7C,COX7A1,COX7A2

Table S4 The GO enrichment of heart DEGs related to lipid metabolism

KEGG	Description	Gene ratio	Gene number	False discorvery rate	Genes
ssc04714	Thermogenesis	5/200	5	0.002	COX17,CYP7A1,COX5B,PPARG,PPARA
ssc05014	Amyotrophic lateral sclerosis	4/306	4	0.002	CYP7A1,COX5B,GPX3,TBK1
ssc04932	Non-alcoholic fatty liver disease	4/124	4	0.003	CYP7A1,COX5B,PPARA,PPARG
ssc05202	Transcriptional misregulation in cancer	4/158	4	0.005	RXRG,PPARG,PPARA,CEBPB
ssc00190	Oxidative phosphorylation	3/115	3	0.003	COX17,CYP7A1,COX5B
ssc03320	PPAR signaling pathway	3/63	3	0.001	RXRG,PPARA,PPARG
ssc04920	Adipocytokine signaling pathway	3/64	3	0.026	RXRG,PPARA,PPARG
ssc04931	Insulin resistance	3/97	3	0.041	PPARA,PPARG,PPP1CB
ssc04260	Cardiac muscle contraction	2/69	2	0.026	CYP7A1,COX5B
ssc04657	IL-17 signaling pathway	2/79	2	0.029	CEBPB,TBK1

Table S5 The blood biochemical criterions related to lipid metabolism

Blood	WZS1	LW1	P value
ALT	49.31±4.90	62.22±18.55	0.135
AST	74.51±21.02	63.42±24.22	0.065
ALP	240.47±21.47[a]	310.36±56.04[b]	0.011
γ-GT	48.82±4.19 [a]	38.99±10.03 [b]	0.031
DBIL	9.79±6.33 [a]	5.06±2.09 [b]	0.045
TBIL	22.43±4.44 [a]	9.37±3.85 [b]	0.0001
TBA	40.78±20.12	31.35±21.05	0.331

TG	0.67±0.27	0.59±0.31	0.383
CHO	2.42±0.30	2.39±0.32	0.101
HDL	0.68±0.22	0.72±0.14	0.447
LDL	1.48±0.28 [a]	1.17±0.09 [b]	0.006

Table S6 The lipid metabolites in liver of WZS and LW pigs

	Formula	polarity	Class	log2 FC	P value	ROC	VIP	WZS pigs mean	LW pigs mean
1	C14 H28 O3	Neg	Fatty Acyls	3.56	1.64 E-06	1	2.41	49624863.82	3377070.31
2	C25 H41 O7 P	Neg	Glycerophospholipids	1.48	2.87 E-05	1	2.09	1885983.61	528872.87
3	C24 H44 N O9 P	Neg	Glycerophospholipids	1.29	0.000147	1	1.58	56225051.08	19594807.14
4	C21 H39 O7 P	Neg	Glycerophospholipids	1.77	0.00019	1	1.61	9839220.95	2419419.31
5	C20 H42 N O7 P	Neg	Glycerophospholipids	1.45	0.000285	1	1.23	7957343.42	3410722.16
6	C28 H48 N O9 P	Neg	Glycerophospholipids	2.05	0.00041	0.98	1.63	47117565.49	5223030.03
7	C26 H46 N O9 P	Neg	Glycerophospholipids	1.61	0.000941	0.96	1.64	26079181.95	6944844.14
8	C25 H43 O7 P	Neg	Glycerophospholipids	1.81	0.001022	0.96	1.79	3648264.44	589082.87
9	C19 H40 N O7 P	Neg	Glycerophospholipids	1.49	0.001024	1	1.05	12786932.23	5099204.78
10	C24 H39 Na O5	Neg	Steroids and steroid derivatives	1.96	0.001801	1	1.82	220691418.01	53266482.63
11	C26 H48 N O7 P	Neg	Glycerophospholipids	1.94	0.002153	1	1.18	79496191.18	21697488.17
12	C6 H10 O4	Neg	Fatty Acyls	-1.79	0.002278	0.87	1.63	50608515.61	235019684.30

13	C28 H46 N O9 P	Neg	Glycerophospholipids	1.73	0.002379	1	1.43	72412272.88	15922882.64
14	C6 H12 O2	Neg	Fatty Acyls	-1.47	0.002958	0.91	1.40	1206026.47	4218564.69
15	C5 H6 O4	Neg	Fatty Acyls	-1.21	0.003167	0.87	1.93	19358524.97	35217686.70
16	C20 H32 O4	Neg	Fatty Acyls	1.58	0.003182	0.96	1.71	2624460.85	1126120.49
17	C18 H22 O2	Neg	Steroids and steroid derivatives	-2.01	0.003524	0.92	1.72	492437.36	2341192.20
18	C18 H28 O3	Neg	Fatty Acyls	1.41	0.003785	0.92	1.67	2506496.19	1222174.89
19	C28 H49 O9 P	Neg	Glycerophospholipids	1.34	0.00403	0.87	1.83	2946181.16	1409353.91
20	C7 H12 O4	Neg	Fatty Acyls	-1.55	0.004635	0.85	1.29	27247443.83	103238940.34
21	C21 H40 Na O7 P	Neg	Glycerophospholipids	1.12	0.005717	0.96	1.52	1749741.61	845458.95
22	C11 H21 N O4	Neg	Fatty Acyls	-1.83	0.006084	0.85	1.49	964321.37	4630560.11
23	C22 H44 N O7 P	Neg	Glycerophospholipids	1.67	0.007238	1	1.23	19850251.39	6225980.49
24	C21 H44 N O7 P	Neg	Glycerophospholipids	1.24	0.009014	0.96	1.06	3669034.32	1551942.10
25	C20 H28 O2	Neg	Prenol lipids	-1.32	0.010195	0.77	1.28	1887633.91	6252497.44
26	C18 H32 O3	Neg	Fatty Acyls	1.26	0.011218	0.87	1.52	1517089.82	735205.32
27	C5 H8 O4	Neg	Fatty Acyls	-1.52	0.014087	0.81	1.42	30842703.35	117873912.41
28	C25 H48 N O7 P	Neg	Glycerophospholipids	1.88	0.014516	0.92	1.63	6002472.01	1810712.03
29	C23 H44 N O7 P	Neg	Glycerophospholipids	0.61	0.017335	0.88	1.27	483036774.94	343054384.16
30	C8 H14 O4	Neg	Fatty Acyls	-0.94	0.018353	0.79	1.31	35268000.84	85059388.53

31	C20 H32 O6	Neg	Fatty Acyls	-0.88	0.030442	0.74	1.36	790979.99	1837689.16
32	C20 H34 O4	Neg	Fatty Acyls	0.80	0.032192	0.83	1.14	3853156.16	2175036.69
33	C20 H30 O5	Neg	Fatty Acyls	-1.54	0.033797	0.70	1.26	711172.05	2879517.85
34	C25 H50 N O7 P	Neg	Glycerophospholipids	1.85	0.035912	0.88	1.23	3407842.73	948421.23
35	C6 H12 O4	Neg	Fatty Acyls	-2.55	0.036712	0.79	1.12	5962937.03	50275490.43
36	C14 H28 O3	Neg	Fatty Acyls	-0.58	0.038673	0.75	1.20	2404219.02	3555897.94
37	C20 H32 O5	Neg	Fatty Acyls	-0.96	0.040901	0.68	1.00	5892707.39	14862215.84
38	C20 H32 O5	Neg	Fatty Acyls	-1.46	0.042898	0.77	1.14	811563.37	3081121.41
39	C24 H38 O4	Neg	Steroids and steroid derivatives	-2.45	0.048093	0.70	1.18	8870052.63	71080126.81
40	C8 H17 N O2	Pos	Fatty Acyls	-3.59	4.26E-09	1	2.282716	1025372.60	14464952.93
41	C26 H44 O9	Pos	Fatty Acyls	3.12	0.000175	1	2.245029	2147501.37	244927.90
42	C15 H30 O4	Pos	Glycerolipids	0.87	0.000255	1	1.488574	6671755.04	3912849.59
43	C19 H28 O2	Pos	Steroids and steroid derivatives	1.13	0.00052	0.944444	1.111752	4735945.72	1977799.46
44	C18 H34 O2	Pos	Fatty Acyls	1.65	0.001291	0.981481	2.004499	10817717.33	3709949.79
45	C14 H20 O2	Pos	Prenol lipids	-5.21	0.001447	1	1.651662	319031.19	17519219.40
46	C18 H30 O6	Pos	Fatty Acyls	-0.68	0.003952	0.925926	1.372181	74836.83	108549.21
47	C23 H45 N O4	Pos	Fatty Acyls	-2.12	0.006735	0.87037	1.351521	379614.80	2275950.44
48	C11 H14 O2	Pos	Fatty Acyls	-2.28	0.006902	0.796296	1.099465	1192124.69	8208386.77
49	C19 H30 O2	Pos	Steroids and steroid derivatives	-2.07	0.007775	0.833333	1.332542	310154.53	1804454.72
50	C26 H30 O8	Pos	Prenol lipids	-0.94	0.009933	0.907407	1.390238	3389404.13	6992103.36
51	C5 H8 O5	Pos	Fatty Acyls	0.96	0.010614	0.87037	1.075867	3527793.24	1523400.61

52	C21 H30 O3	Pos	Steroids and steroid derivatives	-1.48	0.011029	0.777778	1.415626	1355034.74	5044961.59
53	C19 H26 O4	Pos	Prenol lipids	-1.20	0.011046	0.833333	1.158207	506934.43	1463875.45
54	C26 H43 N O6	Pos	Steroids and steroid derivatives	1.48	0.01133	0.888889	1.470492	3613437893.55	1167887647.73
55	C19 H34 O4	Pos	Fatty Acyls	-3.73	0.02169	0.722222	1.269718	1211196.04	23864190.47
56	C5 H8 O3	Pos	Fatty Acyls	-0.77	0.022438	0.851852	1.070717	4471742.03	8782442.73
57	C18 H26 O2	Pos	Steroids and steroid derivatives	-0.60	0.045808	0.777778	1.198547	613817.16	1028490.00
58	C10 H14 O	Pos	Prenol lipids	-1.44	0.047328	0.666667	1.175689	2366200.50	8829077.88

Table S7 The lipid metabolites in heart of WZS and LW pigs

	Formula	polarity	Class	log2 FC	P value	ROC	VIP	WZS pigs mean	LW pigs mean
1	C8 H20 N O6 P	Neg	Glycerophospholipids	-6.509838	3.29E-08	1	1.547664	6286889.22	572917673.11
2	C14 H28 O2	Neg	Fatty Acyls	-3.931732	6.42E-08	1	1.439857	6286889.22	572917673.12
3	C26 H45 N O7 S	Neg	Steroids and steroid derivatives	-6.428262	9.74E-08	1	1.568565	732384.88	11176568.86
4	C12 H22 O12	Neg	Fatty Acyls	-5.623281	1.62E-06	1	1.428579	1783368.79	153582228.40
5	C20 H32 O5	Neg	Fatty Acyls	-2.485117	2.74E-06	1	1.170948	7429200.78	366200110.85
6	C5 H12 O7 P2	Neg	Prenol lipids	-2.351924	3.12E-05	1	1.29665	1294633402.39	7002380.34
7	C24 H48 O2	Neg	Fatty Acyls	-2.312587	3.6E-05	1	1.435646	5480620.97	27978824.02
8	C26 H45 N	Neg	Steroids and steroid derivatives	-4.1377	7.07E-05	1	1.434252	2338847.16	11618759.99

	O8 S2			41					
9	C20 H28 O2	Neg	Prenol lipids	-2.224301	8.63E-05	1	1.027713	21407617.00	376836125.45
10	C21 H30 O4	Neg	Steroids and steroid derivatives	-2.388007	0.00012	1	1.39674	28112500.79	131365321.06
11	C21 H28 O5	Neg	Steroids and steroid derivatives	-2.290558	0.000122	1	1.017423	2663234.86	13940272.03
12	C20 H32 O3	Neg	Fatty Acyls	-1.672225	0.000148	1	1.264363	41042545.03	200798749.97
13	C26 H43 N O6	Neg	Steroids and steroid derivatives	-4.62724	0.000211	1	1.318152	177566604.56	565915075.80
14	C20 H30 O4	Neg	Fatty Acyls	2.66236	0.000214	1	1.249732	6729156.07	166302431.27
15	C18 H24 O2	Neg	Steroids and steroid derivatives	6.1258	0.000474	1	1.499838	491835656.56	7043226.87
16	C12 H23 N O4	Neg	Fatty Acyls	8.6034	0.000864	1	1.694279	491835656.56	7043226.87
17	C22 H32 O4	Neg	Fatty Acyls	-1.929062	0.000967	1	1.065469	1002869131.60	2578480.45
18	C7 H12 O5	Neg	Fatty Acyls	4.33889	0.001399	1	1.104496	42739429.74	162754998.62
19	C18 H34 O2	Neg	Fatty Acyls	-4.110763	0.002115	1	1.550354	132251041.15	6535282.99
20	C27 H44 O3	Neg	Steroids and steroid derivatives	-3.013626	0.002192	0.972222	1.410517	1178463309.00	20360066035.77
21	C20 H30 O5	Neg	Fatty Acyls	5.18266	0.002569	1	1.550762	504866.70	4077261.27
22	C18 H24 O3	Neg	Steroids and steroid derivatives	-2.058512	0.003791	0.972222	1.161853	24810416.41	683120.78
23	C26 H43 N O5	Neg	Steroids and steroid derivatives	-3.027085	0.004461	1	1.043122	2587203.94	10777161.35
24	C22 H36 O2	Neg	Fatty Acyls	-3.7698	0.004511	1	1.276078	52706447.44	429642339.04

				18					
25	C24 H40 O4	Neg	Steroids and steroid derivatives	2.75449	0.00456	0.972222	1.168107	334236411.24	4559130516.86
26	C22 H34 O2	Neg	Fatty Acyls	-3.34598	0.005892	0.972222	1.305131	392980181.59	58235425.91
27	C6 H12 O3	Neg	Fatty Acyls	5.00704	0.007086	1	1.514968	286526595.74	2913435562.19
28	C22 H40 O2	Neg	Fatty Acyls	-2.417546	0.007506	0.944444	1.103751	26002437500.43	808619698.87
29	C20 H32 O2	Neg	Fatty Acyls	-2.247381	0.009349	0.944444	1.271281	5238948.40	27989686.70
30	C20 H34 O5	Neg	Fatty Acyls	-0.924871	0.009692	1	1.011228	10782300925.35	51196543220.03
31	C20 H32 O4	Neg	Fatty Acyls	-2.387433	0.010435	0.944444	1.332209	9325536.65	17704665.28
32	C20 H30 O	Neg	Prenol lipids	-2.553947	0.011349	0.972222	1.331573	4156554.89	21748157.10
33	C10 H19 N O4	Neg	Fatty Acyls	4.6474	0.01196	1	1.397647	5181321.95	30426732.64
34	C18 H22 O2	Neg	Steroids and steroid derivatives	2.68196	0.019388	0.916667	1.039422	11857773.79	685924.19
35	C5 H10 O3	Neg	Fatty Acyls	2.73061	0.021047	0.944444	1.192157	208633625.81	32511160.65
36	C26 H52 N O7 P	Pos	Glycerophospholipids	-6.130379	5.99E-07	1	1.784823	3943134.83	276229148.20
37	C19 H24 O2	Pos	Steroids and steroid derivatives	-1.918373	2E-06	1	1.403491	29507380.11	111536850.15
38	C7 H12 O4	Pos	Fatty Acyls	-4.749081	1.15E-05	1	1.536886	27979791.23	752419899.87
39	C22 H42 O2	Pos	Fatty Acyls	-0.906934	2.57E-05	1	1.285989	1493351.14	2800117.16
40	C32 H48 O5	Pos	Prenol lipids	-3.0682	3.68E-05	1	1.542174	13015766.94	109170585.68

				52					
41	C25 H36 O8	Pos	Steroids and steroid derivatives	-3.560997	3.85E-05	1	1.280447	23741890.16	280209175.65
42	C23 H39 N O2	Pos	Fatty Acyls	3.83867	6.08E-05	1	1.211238	32580493.19	2277214.07
43	C6 H6 O4	Pos	Fatty Acyls	-2.79462	6.58E-05	1	1.201703	3777771.91	26211997.10
44	C18 H30 O3	Pos	Fatty Acyls	3.78274	6.67E-05	1	1.420333	173536014.41	12608719.64
45	C39 H79 N2 O6 P	Pos	Sphingolipids	-1.46915	7.25E-05	1	1.040075	423574.60	1172703.15
46	C20 H28 O	Pos	Prenol lipids	-1.794798	7.68E-05	1	1.156227	37867684.86	131388329.85
47	C21 H32 O2	Pos	Steroids and steroid derivatives	-2.036804	8.4E-05	1	1.023649	672352.80	2758902.59
48	C6 H10 O4	Pos	Fatty Acyls	1.65977	0.000101	1	1.677863	623560844.13	197350675.65
49	C26 H30 O8	Pos	Prenol lipids	3.53781	0.000181	1	1.277486	174355059.75	15012315.32
50	C23 H45 N O4	Pos	Fatty Acyls	-4.931748	0.00025	1	1.345633	63984690.43	1952900541.29
51	C20 H30 O2	Pos	Fatty Acyls	-1.281765	0.000278	1	1.168012	16968632.01	41256893.43
52	C8 H16 O2	Pos	Fatty Acyls	-1.198291	0.000295	1	1.078782	6354355415.36	14581189691.53
53	C20 H24 O2	Pos	Steroids and steroid derivatives	-2.792005	0.000637	1	1.626115	770246.08	5334659.37
54	C21 H30 O3	Pos	Steroids and steroid derivatives	-3.526569	0.000553	1	1.329125	44997825.01	518554557.87
55	C18 H35 N O	Pos	Fatty Acyls	-3.790769	0.001104	1	1.570677	156830017.30	2170522908.49
5	C18	Pos	Fatty Acyls	-	0.00	1	1.15	305995412.4	983616500.

6	H30 O2			1.684586	1204		8104	1	44
57	C26 H43 N O4	Pos	Steroids and steroid derivatives	8.81518	0.001322	1	1.395546	1558354861.92	3459642.89
58	C23 H34 O5	Pos	Fatty Acyls	-2.453244	0.001455	0.972222	1.103072	161380259.35	883792516.70
59	C16 H26 O7	Pos	Fatty Acyls	-1.443725	0.002723	0.944444	1.216288	18316793.47	49825752.62
60	C20 H41 N O4 S	Pos	Fatty Acyls	-2.770253	0.003368	0.944444	1.173395	1441565.28	9834753.83
61	C10 H15 N O	Pos	Prenol lipids	2.92256	0.003665	1	1.035984	22837383.67	3012085.72
62	C21 H30 O2	Pos	Steroids and steroid derivatives	-1.777655	0.005982	1	1.405124	4383974.17	15031263.80
63	C21 H30 O5	Pos	Steroids and steroid derivatives	-1.479861	0.006335	0.944444	1.191793	3299764.40	9203765.39
64	C19 H24 O3	Pos	Steroids and steroid derivatives	-2.791577	0.007105	1	1.249972	3376966.90	23381654.01
65	C20 H38 O2	Pos	Fatty Acyls	-1.51992	0.007786	0.916667	1.298445	1676601.03	4808075.76
66	C21 H38 O4	Pos	Fatty Acyls	-3.812438	0.008082	1	1.436664	139960146.82	1966358207.39
67	C18 H37 N O4 S	Pos	Fatty Acyls	-3.021853	0.009261	0.972222	1.334979	28553105.42	231911187.94
68	C23 H40 O3	Pos	Endocannabinoids	-3.078634	0.010512	1	1.302651	1158597236.70	9787995700.72
69	C9 H18 O2	Pos	Fatty Acyls	2.52648	0.011948	0.888889	1.195767	40209164.07	6978750.20
70	C16 H33 N O	Pos	Fatty Acyls	-2.124212	0.013269	0.972222	1.34681	5867962584.02	25582255998.23
7	C21	Pos	Glycerolipids	-	0.02	0.86	1.10	66521647.72	263566811.

1	H40 O4			1.986273	0748	1111	8831		84
72	C17 H34 O2	Pos	Fatty Acyls	-1.861858	0.024611	1	1.24284	8440792.34	30680219.24
73	C24 H50 N O7 P	Pos	Glycerophospholipids	-1.457392	0.029348	0.861111	1.215217	28032350.90	76980066.99

Table S10 The correlations between blood biochemical criterion and liver metabolites

	Formula	ALT	AST	ALP	γ-GT	DBIL	TBIL	TBA	TG	CHO	HDL	LDL
1	C14.H28.O3	-0.243	0.162	-0.520	0.605	0.826	0.805	0.728	0.466	-0.705	-0.264	0.491
2	C25.H41.O7.P	-0.335	0.244	-0.615	0.604	0.779	0.848	0.679	0.430	-0.796	-0.202	0.567
3	C24.H44.N.O9.P	-0.419	0.284	-0.607	0.526	0.740	0.802	0.509	0.434	-0.773	0.022	0.689
4	C21.H39.O7.P	-0.378	0.267	-0.561	0.514	0.679	0.745	0.505	0.410	-0.745	0.031	0.691
5	C20.H42.N.O7.P	-0.181	0.269	-0.416	0.583	0.771	0.755	0.518	0.558	-0.632	0.153	0.556
6	C28.H48.N.O9.P	-0.286	0.155	-0.542	0.558	0.777	0.772	0.726	0.472	-0.699	-0.245	0.568
7	C26.H46.N.O9.P	-0.354	0.303	-0.572	0.515	0.690	0.751	0.529	0.430	-0.707	-0.005	0.765
8	C25.H43.O7.P	-0.205	0.074	-0.501	0.515	0.783	0.727	0.800	0.548	-0.605	-0.324	0.529
9	C19.H40.N.O7.P	-0.258	0.210	-0.344	0.531	0.695	0.678	0.445	0.456	-0.602	0.171	0.611
10	C24.H39.Na.O5	-0.435	0.535	-0.530	0.325	0.079	0.587	-0.018	0.073	-0.796	0.541	0.719
11	C26.H48.N.O7.P	-0.256	0.060	-0.393	0.463	0.709	0.623	0.685	0.477	-0.555	-0.163	0.523
12	C6.H10.O4	0.754	-0.318	0.810	-0.296	-0.469	-0.774	-0.276	-0.196	0.900	-0.059	-0.652
13	C28.H46.N.O9.P	-0.253	0.054	-0.529	0.467	0.792	0.717	0.802	0.541	-0.592	-0.356	0.474
14	C6.H12.O2	0.180	-0.626	0.541	-0.741	-0.632	-0.814	-0.248	-0.089	0.786	-0.016	-0.617
15	C5.H6.O4	0.787	-0.163	0.750	-0.056	-0.391	-0.522	-0.332	-0.158	0.576	0.221	-0.549
16	C20.H32.O4	-0.302	0.202	-0.271	0.470	0.624	0.557	0.244	0.237	-0.542	0.173	0.402
17	C18.H22.O2	0.745	-0.276	0.827	0.037	-0.161	-0.542	0.086	-0.162	0.777	-0.340	-0.511
18	C18.H28.O3	-0.189	-0.100	-0.377	0.408	0.616	0.568	0.816	0.420	-0.485	-0.493	0.299
19	C28.H49.O9.P	0.212	0.712	-0.236	0.549	0.166	0.548	-0.051	0.326	-0.497	0.758	0.533
20	C7.H12.O4	0.269	-0.610	0.551	-0.730	-0.623	-0.820	-0.228	-0.041	0.831	-0.031	-0.635
21	C21.H40.Na.O7.P	-0.249	0.042	-0.433	0.453	0.749	0.662	0.747	0.513	-0.552	-0.257	0.544
22	C11.H21.N.O4	-0.009	-0.648	0.358	-0.850	-0.614	-0.785	-0.250	-0.107	0.712	-0.109	-0.581
23	C22.H44.N.O7.P	-0.145	-0.044	-0.359	0.452	0.747	0.606	0.829	0.577	-0.482	-0.301	0.393

24	C21.H44.N.O7.P	-0.111	0.082	-0.288	0.561	0.730	0.632	0.715	0.436	-0.536	-0.199	0.484
25	C20.H28.O2	0.198	-0.696	0.541	-0.766	-0.573	-0.852	-0.191	-0.083	0.854	-0.173	-0.673
26	C18.H32.O3	-0.322	-0.138	-0.449	0.255	0.709	0.558	0.756	0.594	-0.387	-0.371	0.329
27	C5.H8.O4	0.136	-0.540	0.540	-0.695	-0.611	-0.751	-0.281	-0.159	0.735	0.016	-0.531
28	C25.H48.N.O7.P	-0.164	-0.019	-0.419	0.440	0.806	0.646	0.800	0.661	-0.445	-0.296	0.497
29	C23.H44.N.O7.P	-0.390	-0.021	-0.525	0.259	0.663	0.589	0.657	0.654	-0.465	-0.127	0.553
30	C8.H14.O4	0.175	-0.607	0.538	-0.732	-0.630	-0.804	-0.282	-0.084	0.781	0.025	-0.620
31	C20.H32.O6	0.154	-0.690	0.507	-0.742	-0.547	-0.775	-0.168	0.010	0.762	-0.042	-0.600
32	C20.H34.O4	0.144	0.590	-0.331	0.574	0.607	0.597	0.210	0.257	-0.333	0.017	0.537
33	C20.H30.O5	0.095	-0.756	0.455	-0.870	-0.651	-0.902	-0.231	-0.179	0.797	-0.249	-0.707
34	C25.H50.N.O7.P	-0.158	-0.068	-0.315	0.428	0.761	0.555	0.757	0.539	-0.415	-0.306	0.405
35	C6.H12.O4	0.801	-0.235	0.590	-0.241	-0.349	-0.564	-0.248	0.019	0.664	0.096	-0.539
36	C14.H28.O3.1	-0.243	0.162	-0.520	0.605	0.826	0.805	0.728	0.466	-0.705	-0.264	0.491
37	C20.H32.O5	0.004	-0.726	0.432	-0.789	-0.652	-0.845	-0.215	-0.195	0.708	-0.208	-0.655
38	C20.H32.O5.1	0.004	-0.726	0.432	-0.789	-0.652	-0.845	-0.215	-0.195	0.708	-0.208	-0.655
39	C24.H38.O4	0.165	-0.717	0.552	-0.768	-0.508	-0.863	-0.211	-0.252	0.855	-0.361	-0.679
40	C8.H17.N.O2	0.613	-0.482	0.805	-0.486	-0.537	-0.851	-0.327	-0.263	0.921	-0.115	-0.709
41	C26.H44.O9	-0.308	0.386	-0.500	0.564	0.379	0.709	0.361	0.029	-0.852	-0.011	0.324
42	C15.H30.O4	-0.370	0.266	-0.488	0.498	0.356	0.653	0.488	0.135	-0.779	-0.090	0.648
43	C19.H28.O2	-0.437	0.219	-0.588	0.495	0.703	0.785	0.588	0.370	-0.780	-0.100	0.590
44	C18.H34.O2	-0.160	-0.009	-0.408	0.455	0.747	0.649	0.809	0.504	-0.507	-0.398	0.380
45	C14.H20.O2	0.201	-0.372	0.382	-0.596	-0.456	-0.792	-0.291	-0.351	0.828	-0.425	-0.569
46	C18.H30.O6	-0.100	-0.621	0.328	-0.871	-0.859	-0.831	-0.456	-0.466	0.476	-0.068	-0.544
47	C23.H45.N.O4	0.017	-0.495	0.380	-0.665	-0.494	-0.695	-0.132	-0.092	0.721	-0.156	-0.476
48	C11.H14.O2	0.235	-0.540	0.528	-0.738	-0.587	-0.912	-0.332	-0.310	0.928	-0.311	-0.662
49	C19.H30.O2	0.046	-0.603	0.294	-0.873	-0.560	-0.806	-0.421	-0.308	0.683	-0.255	-0.610
50	C26.H30.O8	0.844	-0.288	0.678	-0.184	-0.363	-0.612	0.006	-0.066	0.754	-0.235	-0.572
51	C5.H8.O5	-0.115	0.449	-0.446	0.757	0.730	0.784	0.428	0.150	-0.680	-0.214	0.489

52	C21.H30.O3	0.003	-0.631	0.423	-0.703	-0.593	-0.793	-0.209	-0.179	0.703	-0.178	-0.612
53	C19.H26.O4	0.303	-0.558	0.708	-0.650	-0.699	-0.903	-0.372	-0.272	0.875	-0.017	-0.667
54	C26.H43.N.O6	-0.474	0.643	-0.595	0.288	0.115	0.627	-0.150	0.130	-0.749	0.661	0.806
55	C19.H34.O4	-0.016	-0.496	0.292	-0.684	-0.495	-0.642	-0.097	0.017	0.651	-0.085	-0.432
56	C5.H8.O3	0.560	0.066	0.331	-0.239	-0.253	-0.467	-0.284	-0.154	0.670	-0.138	-0.285
57	C18.H26.O2	0.016	-0.532	0.169	-0.831	-0.553	-0.635	-0.393	0.090	0.544	0.183	-0.421
58	C10.H14.O	0.145	-0.617	0.462	-0.740	-0.602	-0.795	-0.174	-0.033	0.779	-0.083	-0.589

Table S11 The significance of correlations between blood biochemical criterion and liver metabolites

	Formula	ALT	AST	ALP	γ-GT	DBIL	TBIL	TBA	TG	CHO	HDL	LDL
1	C14.H28.O3	--	--	--	0.0371	0.0009	0.0016	0.0073	--	0.0105	--	--
2	C25.H41.O7.P	--	--	0.0332	0.0377	0.0028	0.0005	0.0152	--	0.0019	--	--
3	C24.H44.N.O9.P	--	--	0.0364	--	0.0060	0.0017	--	--	0.0032	--	0.0133
4	C21.H39.O7.P	--	--	--	--	0.0152	0.0054	--	--	0.0054	--	0.0128
5	C20.H42.N.O7.P	--	--	--	0.0467	0.0034	0.0045	--	--	0.0275	--	--
6	C28.H48.N.O9.P	--	--	--	--	0.0030	0.0033	0.0076	--	0.0114	--	--
7	C26.H46.N.O9.P	--	--	--	--	0.0131	0.0049	--	--	0.0102	--	0.0037
8	C25.H43.O7.P	--	--	--	--	0.0026	0.0074	0.0018	--	0.0373	--	--
9	C19.H40.N.O7.P	--	--	--	--	0.0120	0.0154	--	--	0.0383	--	0.0347
10	C24.H39.Na.O5	--	--	--	--	--	0.0446	--	--	0.0020	--	0.0084
11	C26.H48.N.O7.P	--	--	--	--	0.0098	0.0304	0.0140	--	--	--	--

12	C6.H10.O4	0.0046	--	0.0014	--	--	0.0031	--	--	0.0001	--	0.0215
13	C28.H46.N.O9.P	--	--	--	--	0.0021	0.0087	0.0017	--	0.0424	--	--
14	C6.H12.O2	--	0.0296	--	0.0059	0.0275	0.0013	--	--	0.0024	--	0.0326
15	C5.H6.O4	0.0024	--	0.0050	--	--	--	--	--	--	--	--
16	C20.H32.O4	--	--	--	--	0.0300	--	--	--	--	--	--
17	C18.H22.O2	0.0054	--	0.0009	--	--	--	--	--	0.0029	--	--
18	C18.H28.O3	--	--	--	--	0.0330	--	0.0012	--	--	--	--
19	C28.H49.O9.P	--	0.0094	--	--	--	--	--	--	--	0.0042	--
20	C7.H12.O4	--	0.0354	--	0.0070	0.0303	0.0011	--	--	0.0008	--	0.0266
21	C21.H40.Na.O7.P	--	--	--	--	0.0051	0.0190	0.0052	--	--	--	--
22	C11.H21.N.O4	--	0.0227	--	0.0005	0.0336	0.0025	--	--	0.0094	--	0.0477
23	C22.H44.N.O7.P	--	--	--	--	0.0052	0.0369	0.0008	0.0497	--	--	--
24	C21.H44.N.O7.P	--	--	--	--	0.0070	0.0273	0.0090	--	--	--	--
25	C20.H28.O2	--	0.0120	--	0.0037	--	0.0004	--	--	0.0004	--	0.0166
26	C18.H32.O3	--	--	--	--	0.0099	--	0.0044	0.0415	--	--	--
27	C5.H8.O4	--	--	--	0.0122	0.0348	0.0049	--	--	0.0064	--	--
28	C25.H48.N.O7.P	--	--	--	--	0.0016	0.0233	0.0018	0.0191	--	--	--
29	C23.H44.N.O7.P	--	--	--	--	0.0188	0.0440	0.0203	0.0211	--	--	--
30	C8.H14.O4	--	0.0364	--	0.0067	0.0282	0.0016	--	--	0.0027	--	0.0314
31	C20.H32.O6	--	0.0130	--	0.0057	--	0.0031	--	--	0.0040	--	0.0390
32	C20.H34.O4	--	0.0433	--	--	0.0363	0.0405	--	--	--	--	--
33	C20.H30.O5	--	0.0044	--	0.0002	0.0219	0.0001	--	--	0.0019	--	0.0101

34	C25.H50.N.O7.P	--	--	--	--	0.0040	--	0.0044	--	--	--	--
35	C6.H12.O4	0.0017	--	0.0434	--	--	--	--	--	0.0186	--	--
36	C14.H28.O3.1	--	--	--	0.0371	0.0009	0.0016	0.0073	--	0.0105	--	--
37	C20.H32.O5	--	0.0075	--	0.0023	0.0215	0.0005	--	--	0.0100	--	0.0209
38	C20.H32.O5.1	--	0.0075	--	0.0023	0.0215	0.0005	--	--	0.0100	--	0.0209
39	C24.H38.O4	--	0.0086	--	0.0035	--	0.0003	--	--	0.0004	--	0.0152
40	C8.H17.N.O2	0.0339	--	0.0016	--	--	0.0005	--	--	0.0000	--	0.0098
41	C26.H44.O9	--	--	--	--	--	0.0099	--	--	0.0004	--	--
42	C15.H30.O4	--	--	--	--	--	0.0212	--	--	0.0028	--	0.0227
43	C19.H28.O2	--	--	0.0443	--	0.0108	0.0025	0.0445	--	0.0028	--	0.0434
44	C18.H34.O2	--	--	--	--	0.0053	0.0223	0.0015	--	--	--	--
45	C14.H20.O2	--	--	--	0.0409	--	0.0021	--	--	0.0009	--	--
46	C18.H30.O6	--	0.0310	--	0.0002	0.0003	0.0008	--	--	--	--	--
47	C23.H45.N.O4	--	--	--	0.0183	--	0.0121	--	--	0.0081	--	--
48	C11.H14.O2	--	--	--	0.0061	0.0449	0.0000	--	--	0.0000	--	0.0191
49	C19.H30.O2	--	0.0379	--	0.0002	--	0.0015	--	--	0.0143	--	0.0351
50	C26.H30.O8	0.0006	--	0.0155	--	--	0.0344	--	--	0.0046	--	--
51	C5.H8.O5	--	--	--	0.0044	0.0070	0.0025	--	--	0.0149	--	--
52	C21.H30.O3	--	0.0277	--	0.0107	0.0423	0.0021	--	--	0.0107	--	0.0344
53	C19.H26.O4	--	--	0.0099	0.0222	0.0114	0.0001	--	--	0.0002	--	0.0177
54	C26.H43.N.O6	--	0.0242	0.0411	--	--	0.0292	--	--	0.0051	0.0194	0.0015
55	C19.H34.O4	--	--	--	0.0141	--	0.0244	--	--	0.0219	--	--

56	C5.H8.O3	--	--	--	--	--	--	--	--	0.0172	--	--
57	C18.H26.O2	--	--	--	0.0008	--	0.0265	--	--	--	--	--
58	C10.H14.O	--	0.0327	--	0.0059	0.0385	0.0020	--	--	0.0028	--	0.0437

Note: The symbol "—" meant *p* value ≥0.05.

Table S12 The correlation between blood biochemical criterion and heart metabolites

	Formula	ALT	AST	ALP	γ-GT	DBIL	TBIL	TBA	TG	CHO	HDL	LDL
1	C8 H20 N O6 P	0.603	-0.367	0.700	-0.386	-0.488	-0.758	-0.127	-0.031	0.912	-0.071	-0.607
2	C14 H28 O2	0.297	-0.566	0.661	-0.589	-0.578	-0.865	-0.179	-0.215	0.900	-0.225	-0.665
3	C26 H45 N O7 S	0.445	-0.586	0.625	-0.709	-0.597	-0.898	-0.305	-0.153	0.926	-0.145	-0.717
4	C12 H22 O12	0.349	-0.417	0.635	-0.567	-0.526	-0.846	-0.266	-0.286	0.947	-0.249	-0.607
5	C20 H32 O5	0.379	-0.643	0.812	-0.563	-0.573	-0.868	-0.264	-0.244	0.876	-0.091	-0.709
6	C5 H12 O7 P2	0.442	-0.322	0.649	-0.477	-0.485	-0.818	-0.279	-0.261	0.946	-0.192	-0.567
7	C24 H48 O2	0.573	-0.450	0.867	-0.337	-0.421	-0.797	-0.206	-0.259	0.937	-0.186	-0.658
8	C26 H45 N O8 S2	0.086	-0.755	0.544	-0.783	-0.609	-0.917	-0.314	-0.330	0.798	-0.293	-0.736
9	C20 H28 O2	0.822	-0.067	0.617	-0.168	-0.293	-0.589	-0.148	0.015	0.846	-0.033	-0.433
10	C21 H30 O4	0.478	-0.560	0.519	-0.592	-0.462	-0.775	-0.293	-0.092	0.757	-0.127	-0.693
11	C21 H28 O5	0.437	-0.531	0.630	-0.555	-0.610	-0.873	-0.183	-0.153	0.875	-0.184	-0.634
12	C20 H32 O3	0.664	-0.152	0.612	-0.350	-0.398	-0.667	-0.237	-0.033	0.881	0.022	-0.426
13	C26 H43 N O6	0.239	-0.398	0.385	-0.544	-0.483	-0.642	-0.036	0.108	0.724	-0.040	-0.465
14	C20 H30 O4	-0.349	0.426	-0.469	0.515	0.264	0.658	0.271	0.020	-0.875	0.208	0.463
15	C18 H24 O2	-0.408	0.588	-0.680	0.414	0.308	0.708	0.037	0.118	-0.726	0.200	0.671
16	C12 H23 N O4	-0.367	0.532	-0.511	0.142	-0.101	0.358	-0.155	0.001	-0.518	0.410	0.806
17	C22 H32 O4	0.340	-0.474	0.660	-0.594	-0.602	-0.910	-0.370	-0.374	0.927	-0.229	-0.657
18	C7 H12 O5	-0.368	0.483	-0.370	0.449	0.276	0.540	-0.024	-0.019	-0.727	0.452	0.587
19	C18 H34 O2	0.329	-0.719	0.758	-0.631	-0.588	-0.881	-0.267	-0.223	0.845	-0.111	-0.733
20	C27 H44 O3	0.011	-0.748	0.372	-0.827	-0.542	-0.731	-0.333	-0.160	0.562	-0.071	-0.598
21	C20 H30 O5	-0.303	0.456	-0.317	0.385	0.038	0.474	-0.064	-0.200	-0.723	0.368	0.219
22	C18 H24 O3	0.881	0.017	0.601	0.047	-0.152	-0.437	0.014	0.099	0.753	-0.075	-0.378
23	C26 H43 N O5	0.682	-0.211	0.552	-0.252	-0.329	-0.522	0.036	0.272	0.770	0.088	-0.424
24	C22 H36 O2	0.626	-0.362	0.889	-0.196	-0.396	-0.701	-0.149	-0.190	0.863	-0.074	-0.587
25	C24 H40 O4	0.020	-0.041	-0.317	0.457	0.580	0.550	0.914	0.496	-0.461	-0.439	0.265
26	C22 H34 O2	0.632	-0.275	0.845	-0.102	-0.297	-0.647	-0.114	-0.275	0.848	-0.239	-0.557
27	C6 H12 O3	-0.437	0.592	-0.602	0.203	0.084	0.525	-0.178	0.157	-0.594	0.539	0.892
28	C22 H40 O2	0.345	-0.563	0.842	-0.346	-0.397	-0.681	-0.247	-0.187	0.702	0.092	-0.543
29	C20 H32 O2	0.523	-0.377	0.840	-0.150	-0.260	-0.660	-0.089	-0.287	0.847	-0.305	-0.598
30	C20 H34 O5	0.571	-0.491	0.566	-0.437	-0.161	-0.643	0.018	0.159	0.883	-0.249	-0.473

31	C20 H32 O4	0.511	-0.396	0.825	-0.194	-0.291	-0.674	-0.066	-0.264	0.858	-0.306	-0.624
32	C20 H30 O	0.494	-0.407	0.854	-0.157	-0.294	-0.674	-0.089	-0.325	0.827	-0.315	-0.614
33	C10 H19 N O4	-0.213	0.149	-0.517	0.266	0.624	0.642	0.398	0.615	-0.364	-0.050	0.495
34	C18 H22 O2	-0.355	0.570	-0.456	0.254	-0.114	0.361	-0.207	-0.224	-0.590	0.321	0.586
35	C5 H10 O3	-0.426	0.591	-0.603	0.173	0.060	0.500	-0.206	0.125	-0.544	0.483	0.840
36	C26 H52 N O7 P	0.553	-0.479	0.739	-0.539	-0.557	-0.879	-0.257	-0.187	0.981	-0.161	-0.700
37	C19 H24 O2	0.478	-0.519	0.570	-0.623	-0.574	-0.885	-0.257	-0.152	0.900	-0.212	-0.668
38	C7 H12 O4	0.301	-0.241	0.361	-0.501	-0.416	-0.723	-0.215	-0.217	0.834	-0.339	-0.497
39	C22 H42 O2	0.423	-0.628	0.556	-0.701	-0.494	-0.857	-0.247	-0.125	0.863	-0.229	-0.664
40	C32 H48 O5	0.172	-0.757	0.414	-0.796	-0.526	-0.804	-0.289	-0.166	0.678	-0.228	-0.705
41	C25 H36 O8	0.462	-0.442	0.525	-0.548	-0.535	-0.826	-0.206	-0.106	0.870	-0.199	-0.640
42	C23 H39 N O2	-0.289	0.279	-0.609	0.592	0.808	0.874	0.549	0.440	-0.719	-0.180	0.454
43	C6 H6 O4	0.385	-0.397	0.555	-0.608	-0.546	-0.876	-0.320	-0.270	0.941	-0.249	-0.610
44	C18 H30 O3	-0.388	0.499	-0.433	0.379	0.022	0.530	-0.004	-0.142	-0.814	0.391	0.437
45	C39 H79 N2 O6 P	0.210	-0.702	0.682	-0.659	-0.631	-0.878	-0.361	-0.309	0.784	-0.108	-0.735
46	C20 H28 O	0.434	-0.462	0.401	-0.715	-0.480	-0.781	-0.281	0.031	0.859	-0.097	-0.569
47	C21 H32 O2	0.305	-0.728	0.655	-0.673	-0.515	-0.899	-0.161	-0.181	0.917	-0.327	-0.746
48	C6 H10 O4	-0.412	0.730	-0.556	0.633	0.369	0.806	-0.014	-0.011	-0.912	0.452	0.656
49	C26 H30 O8	-0.325	0.449	-0.414	0.532	0.351	0.658	0.105	-0.091	-0.796	0.172	0.218
50	C23 H45 N O4	0.144	-0.685	0.589	-0.711	-0.583	-0.818	-0.197	-0.164	0.793	-0.145	-0.638
51	C20 H30 O2	0.682	-0.305	0.565	-0.354	-0.258	-0.675	-0.057	-0.041	0.897	-0.336	-0.559
52	C8 H16 O2	0.446	-0.471	0.724	-0.597	-0.627	-0.926	-0.341	-0.319	0.937	-0.131	-0.699
53	C20 H24 O2	-0.075	-0.722	0.269	-0.921	-0.590	-0.816	-0.261	-0.147	0.686	-0.236	-0.608
54	C21 H30 O3	0.403	-0.614	0.668	-0.626	-0.533	-0.855	-0.398	-0.321	0.797	-0.157	-0.700
55	C18 H35 N O	0.210	-0.707	0.662	-0.706	-0.584	-0.909	-0.252	-0.249	0.879	-0.219	-0.712
56	C18 H30 O2	0.300	-0.685	0.400	-0.778	-0.543	-0.829	-0.173	-0.026	0.754	-0.218	-0.620
57	C26 H43 N O4	0.003	-0.184	-0.289	0.374	0.702	0.490	0.887	0.519	-0.294	-0.625	0.165
58	C23 H34 O5	0.412	-0.291	0.490	-0.352	-0.276	-0.635	-0.292	-0.410	0.692	-0.405	-0.473
59	C16 H26 O7	0.374	-0.495	0.559	-0.614	-0.490	-0.823	-0.286	-0.094	0.828	-0.031	-0.591
60	C20 H41 N O4 S	-0.159	-0.550	0.288	-0.708	-0.426	-0.654	-0.125	-0.116	0.639	-0.211	-0.415
61	C10 H15 N O	-0.017	-0.133	-0.314	0.409	0.668	0.512	0.907	0.489	-0.368	-0.591	0.241
62	C21 H30 O2	0.771	-0.193	0.600	-0.215	-0.233	-0.604	-0.185	-0.051	0.851	-0.186	-0.634
63	C21 H30 O5	0.207	-0.429	0.346	-0.752	-0.717	-0.802	-0.254	-0.023	0.724	0.061	-0.417
64	C19 H24 O3	0.512	-0.124	0.741	-0.109	-0.284	-0.584	-0.256	-0.441	0.751	-0.219	-0.432
65	C20 H38 O2	0.402	-0.768	0.494	-0.543	-0.200	-0.694	0.206	0.108	0.839	-0.595	-0.654
66	C21 H38 O4	0.374	-0.610	0.706	-0.521	-0.461	-0.839	-0.347	-0.425	0.801	-0.318	-0.712
67	C18 H37 N O4 S	-0.121	-0.632	0.296	-0.758	-0.434	-0.660	-0.098	-0.067	0.645	-0.228	-0.457
68	C23 H40 O3	0.339	-0.748	0.736	-0.507	-0.469	-0.776	-0.256	-0.249	0.680	-0.133	-0.714
69	C9 H18 O2	-0.255	0.364	-0.344	0.322	-0.069	0.407	0.149	-0.149	-0.694	0.182	0.282
70	C16 H33 N O	0.179	-0.667	0.655	-0.591	-0.418	-0.812	-0.264	-0.207	0.822	-0.166	-0.635
71	C21 H40 O4	0.044	-0.683	0.295	-0.642	-0.336	-0.587	-0.283	-0.138	0.393	-0.099	-0.510
72	C17 H34 O2	0.333	-0.765	0.628	-0.620	-0.317	-0.772	-0.122	-0.013	0.854	-0.260	-0.662
73	C24 H50 N O7 P	-0.087	-0.635	0.136	-0.813	-0.354	-0.622	-0.154	-0.103	0.591	-0.395	-0.576

Table S13 The significance of correlation between blood biochemical criterion and heart metabolites

	Formula	ALT	AST	ALP	γ-GT	DBIL	TBIL	TBA	TG	CHO	HDL	LDL
1	C8 H20 N O6 P	0.0379	--	0.0112	--	--	0.0042	--	--	3.62E-05	--	0.0363
2	C14 H28 O2	--	--	0.0193	0.0437	0.0489	0.0003	--	--	6.57E-05	--	0.0182
3	C26 H45 N O7 S	--	0.0454	0.0299	0.0099	0.0403	0.0001	--	--	1.51E-05	--	0.0087
4	C12 H22 O12	--	--	0.0266	--	--	0.0005	--	--	3.04E-06	--	0.0363
5	C20 H32 O5	--	0.024	0.0013	--	--	0.0002	--	--	0.0002	--	0.0099
6	C5 H12 O7 P2	--	--	0.0225	--	--	0.0012	--	--	3.36E-06	--	--
7	C24 H48 O2	--	--	0.0003	--	--	0.0019	--	--	6.88E-06	--	0.0201
8	C26 H45 N O8 S2	--	0.0045	--	0.0026	0.0354	0.0001	--	--	0.0019	--	0.0064
9	C20 H28 O2	0.001	--	0.0326	--	--	0.0438	--	--	0.0005	--	--
10	C21 H30 O4	--	--	--	0.0425	--	0.0031	--	--	0.0044	--	0.0124
11	C21 H28 O5	--	--	0.0281	--	0.0353	0.0002	--	--	0.0002	--	0.027
12	C20 H32 O3	0.0185	--	0.0343	--	--	0.0177	--	--	0.0002	--	--
13	C26 H43 N O6	--	--	--	--	--	0.0243	--	--	0.0078	--	--
14	C20 H30 O4	--	--	--	--	--	0.02	--	--	0.0002	--	--
15	C18 H24 O2	--	0.0443	0.015	--	--	0.01	--	--	0.0076	--	0.0168
16	C12 H23 N O4	--	--	--	--	--	--	--	--	--	--	0.0016
17	C22 H32 O4	--	--	0.0195	0.0417	0.0385	0.0001	--	--	1.49E-05	--	0.0203
18	C7 H12 O5	--	--	--	--	--	--	--	--	0.0074	--	0.0449
19	C18 H34 O2	--	0.0084	0.0042	0.0279	0.0445	0.0002	--	--	0.0005	--	0.0066
20	C27 H44 O3	--	0.0051	--	0.0009	--	0.0069	--	--	--	--	0.0399
21	C20 H30 O5	--	--	--	--	--	--	--	--	0.0079	--	--
22	C18 H24 O3	0.0002	--	0.0389	--	--	--	--	--	0.0047	--	--
23	C26 H43 N O5	0.0146	--	--	--	--	--	--	--	0.0034	--	--
24	C22 H36 O2	0.0294	--	0.0001	--	--	0.0111	--	--	0.0003	--	0.045
25	C24 H40 O4	--	--	--	--	0.0481	--	3.13E-05	--	--	--	--
26	C22 H34 O2	0.0275	--	0.0005	--	--	0.023	--	--	0.0005	--	--
27	C6 H12 O3	--	0.0426	0.0382	--	--	--	--	--	0.0419	--	0.0001
28	C22 H40 O2	--	--	0.0006	--	--	0.0147	--	--	0.011	--	--
29	C20 H32 O2	--	--	0.0006	--	--	0.0195	--	--	0.0005	--	0.0398
30	C20 H34 O5	--	--	--	--	--	0.024	--	--	0.0001	--	--
31	C20 H32 O4	--	--	0.0009	--	--	0.0162	--	--	0.0004	--	0.0301
32	C20 H30 O	--	--	0.0004	--	--	0.0162	--	--	0.0009	--	0.0338
33	C10 H19 N O4	--	--	--	--	0.0302	0.0244	--	0.0332	--	--	--
34	C18 H22 O2	--	--	--	--	--	--	--	--	0.0434	--	0.0452

35	C5 H10 O3	--	0.0431	0.0378	--	--	--	--	--	--	--	0.0006
36	C26 H52 N O7 P	--	--	0.006	--	--	0.0002	--	--	1.66E-08	--	0.0113
37	C19 H24 O2	--	--	--	0.0305	--	0.0001	--	--	6.63E-05	--	0.0176
38	C7 H12 O4	--	--	--	--	--	0.0079	--	--	0.0008	--	--
39	C22 H42 O2	--	0.0288	--	0.011	--	0.0004	--	--	0.0003	--	0.0185
40	C32 H48 O5	--	0.0044	--	0.0019	--	0.0016	--	--	0.0154	--	0.0104
41	C25 H36 O8	--	--	--	--	--	0.0009	--	--	0.0002	--	0.0251
42	C23 H39 N O2	--	--	0.0355	0.0426	0.0015	0.0002	--	--	0.0085	--	--
43	C6 H6 O4	--	--	--	0.036	--	0.0002	--	--	5.13E-06	--	0.0352
44	C18 H30 O3	--	--	--	--	--	--	--	--	0.0013	--	--
45	C39 H79 N2 O6 P	--	0.0109	0.0145	0.0197	0.0279	0.0002	--	--	0.0025	--	0.0065
46	C20 H28 O	--	--	--	0.009	--	0.0027	--	--	0.0003	--	--
47	C21 H32 O2	--	0.0072	0.0208	0.0165	--	0.0001	--	--	2.72E-05	--	0.0053
48	C6 H10 O4	--	0.007	--	0.0273	--	0.0015	--	--	3.59E-05	--	0.0204
49	C26 H30 O8	--	--	--	--	--	0.0201	--	--	0.0019	--	--
50	C23 H45 N O4	--	0.014	0.0439	0.0096	0.0467	0.0012	--	--	0.0021	--	0.0257
51	C20 H30 O2	0.0146	--	--	--	--	0.0159	--	--	7.81E-05	--	--
52	C8 H16 O2	--	--	0.0077	0.0405	0.029	0	--	--	7.08E-06	--	0.0114
53	C20 H24 O2	--	0.0081	--	0	0.0433	0.0012	--	--	0.0138	--	0.036
54	C21 H30 O3	--	0.0335	0.0177	0.0294	--	0.0004	--	--	0.0019	--	0.0112
55	C18 H35 N O	--	0.0101	0.0191	0.0103	0.0462	0	--	--	0.0002	--	0.0093
56	C18 H30 O2	--	0.014	--	0.0029	--	0.0009	--	--	0.0046	--	0.0314
57	C26 H43 N O4	--	--	--	--	0.011	--	0.0001	--	--	0.0298	--
58	C23 H34 O5	--	--	--	--	--	0.0265	--	--	0.0126	--	--
59	C16 H26 O7	--	--	--	0.0338	--	0.001	--	--	0.0009	--	0.0429
60	C20 H41 N O4 S	--	--	--	0.01	--	0.021	--	--	0.0252	--	--
61	C10 H15 N O	--	--	--	--	0.0175	--	4.60E-05	--	--	0.0431	--
62	C21 H30 O2	0.0033	--	0.0393	--	--	0.0374	--	--	0.0005	--	0.027
63	C21 H30 O5	--	--	--	0.0048	0.0086	0.0017	--	--	0.0077	--	--
64	C19 H24 O3	--	--	0.0058	--	--	0.046	--	--	0.0049	--	--
65	C20 H38 O2	--	0.0035	--	--	--	0.0123	--	--	0.0006	0.0411	0.0211
66	C21 H38 O4	--	0.0353	0.0104	--	--	0.0006	--	--	0.0017	--	0.0094
67	C18 H37 N O4 S	--	0.0274	--	0.0043	--	0.0194	--	--	0.0237	--	--
68	C23 H40 O3	--	0.0051	0.0064	--	--	0.003	--	--	0.0149	--	0.0091
69	C9 H18 O2	--	--	--	--	--	--	--	--	0.0123	--	--
70	C16 H33 N O	--	0.0178	0.0207	0.0428	--	0.0013	--	--	0.001	--	0.0266
71	C21 H40 O4	--	0.0143	--	0.0244	--	0.045	--	--	--	--	--
72	C17 H34 O2	--	0.0037	0.0288	0.0316	--	0.0033	--	--	0.0004	--	0.0191
73	C24 H50 N O7 P	--	0.0265	--	0.0013	--	0.0309	--	--	0.0429	--	0.0498
